# Monoalcohol‐Directed Shape Control in a Surfactant‐Free Gold Nanoplate Synthesis

**DOI:** 10.1002/smtd.70731

**Published:** 2026-05-20

**Authors:** Brendan D. Nieukirk, Shuiyi Zhang, Runze Tang, Kaikui Xu, Matthew R. Rosenberger, Robert A. Hughes, Kristen A. Fichthorn, Svetlana Neretina

**Affiliations:** ^1^ Department of Chemistry and Biochemistry University of Notre Dame Notre Dame Indiana USA; ^2^ Department of Chemical Engineering The Pennsylvania State University University Park Pennsylvania USA; ^3^ College of Engineering University of Notre Dame Notre Dame Indiana USA; ^4^ Department of Physics The Pennsylvania State University University Park Pennsylvania USA

**Keywords:** density functional theory, gold nanoplates, light‐mediated, methanol, monoalcohol

## Abstract

Incorporating shape‐directing additives in colloidal syntheses has proved critical in exerting architectural control over noble metal nanostructures, but these same additives can prove problematic in downstream processing and applications. This deficiency has led to the emergence of a class of synthetic protocols, referred to as surfactant‐free syntheses, that mitigate these issues by using reagents that realize nanoparticle surfaces that are unencumbered by persistent shape‐directing agents. Polyol‐ and monoalcohol‐based growth solutions have proven highly effective in this regard; however, shape‐control in the absence of additives has proved elusive. Herein, a seed‐mediated photochemical synthesis is demonstrated that can form arrays of highly faceted gold nanoplates in a room‐temperature growth solution of HAuCl_4_, water, and a monoalcohol. Wavelength‐dependent characterization and first‐principles density functional theory (DFT) calculations provide insights into the photoredox and shape‐control mechanisms responsible for the unexpected emergence of nanoplates. It is demonstrated that the illumination of the growth solution is the key driver of nanoplate growth. DFT calculations indicate that two‐dimensional growth stems from the {111} surface‐selective co‐adsorption of the monoalcohols and chlorine. Taken together, this work establishes the viability of a shape‐controlled monoalcohol‐based synthesis and advances the mechanistic framework needed to direct nanometal growth trajectories along pathways yielding planar geometries.

## Graphical abstract

Surfactant‐free monoalcohol syntheses are advantageous in that they yield colloidal nanostructures that are free of persistent shape‐directing agents but at the expense of realizing only spherically‐shaped nanoparticles. In this work, this limitation is overcome using a low‐complexity light‐driven synthetic protocol yielding periodic arrays of gold nanoplates.

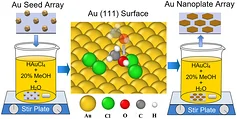

## 1 Introduction

Low‐complexity synthetic protocols realizing functional nanomaterials are those that most often achieve widespread adoption. Such protocols typically use few reagents and are carried out under mild reaction conditions. For the specific case of Au, colloidal syntheses referred to as the Borowskaja–Turkevich−Frens [1], Brust−Schiffrin [2], and polyol [3] methods all fall within this domain. Minimizing the number of reagents, while preferable, inevitably places increased demands on those present since they must take on multiple roles in fulfilling the essential mechanistic requirements of metal precursor, reducing agent, stabilizing/capping agent, and solvent. From this standpoint, polyol syntheses carried out in alkaline media stand out since Au nanoparticles are readily formed [4, 5, 6, 7] where polyols such as glycerol and ethylene glycol, in combination with their reaction products [8, 9], can fulfill all mechanistic roles once the metal precursor is added. Polyol syntheses, especially those carried out at room temperature in glycerol, have gained notice in that they are an important subclass of what are referred to as “surfactant‐free syntheses” [10, 11, 12]. Such syntheses realize nanoparticles that are unencumbered by the negative influences of surfactants that can diminish catalytic activity, obstruct application‐driven functionalization and analyte adhesion, impair electron transfer to adjacent materials, and adversely affect biocompatibility [13, 14, 15, 16, 17, 18, 19]. One of the downsides of the surfactant‐free polyol approach as it relates to Au is that shape control has proved elusive and, with few exceptions [20, 21, 22, 23, 24], has only realized nonspherical nanostructures in significant yields when shape‐directing additives are supplied to the growth solution.

Like polyols, monoalcohols such as methanol (MeOH), ethanol (EtOH), and isopropyl alcohol (i‐PrOH) have long been known for their role in reducing noble metal ions to their zero‐valent state [25]. More recently, Quinson and co‐workers have demonstrated the viability of a surfactant‐free monoalcohol synthesis capable of realizing spherical nanoparticles for a wide variety of noble metals while ensuring colloidal stability [10, 11, 26]. For the case of Au, a typical synthesis proceeds through the injection of aqueous HAuCl_4_ into a room‐temperature solution of a base (*e.g*., NaOH, LiOH), monoalcohol, and H_2_O under constant stirring where the optimized monoalcohol‐to‐H_2_O ratio is in the range of 20% to 30% by volume [5, 27]. Gomes et al. [8] have convincingly argued that it is not the monoalcohol that is the true reducing agent but is instead the alkoxide anions (*e.g*., methoxide, ethoxide) that are derived from them under alkaline conditions. Quinson et al. [28]. further showed that the base significantly contributes to the long‐term stability of the colloid, with LiOH proving most effective. The overall synthetic approach is advantageous in that it (i) offers ease of synthesis, (ii) avoids the post‐synthesis removal of surfactants and high‐viscosity polyols, (iii) realizes nanomaterials with improved catalytic performance, and, to a high degree, (iv) conforms to the principles of green chemistry [27, 29].

The parameter space for surfactant‐free monoalcohol syntheses utilizing MeOH and EtOH has been rigorously mapped out in a series of studies using a highly automated system that performed more than a thousand nanoparticle syntheses [7, 26, 29]. The investigators explored the influence of (i) various reagents and their concentrations, (ii) the order of chemical additions, (iii) light, (iv) temperature, and (v) the purging of the growth solution with N_2_ gas. From the standpoint of the current report, these studies have two important findings. First, they unequivocally demonstrate that the synthesis can have a photochemical component even when carried out under ambient light [7, 29]. Additionally, they showed that shorter wavelength illumination is more impactful where the influence is most prominent in the early stages of the reaction. Second, although these thorough investigations proved successful in defining regions of the parameter space able to exert control over nanoparticle size, monodispersity, and colloidal stability, nonspherical structures were only formed at low yields as diversely shaped minority products [27]. Shape control has, hence, become one of the most challenging pursuits in surfactant‐free syntheses [10].

The synthesis of Au nanoplates has been heavily reliant on the use of surfactants or halides as shape‐directing agents [30−33]. A subset of these syntheses has utilized plasmon‐mediated growth modes [34] to forward the room‐temperature growth of nanoplates both as colloids [35−38] and as substrate‐supported structures [39, 40]. In a recent report [41], we demonstrated an alternate photochemical pathway for forming periodic arrays of Au nanoplates that differ from those that are plasmon‐mediated in that all of the required photochemistry occurs within the growth solution as opposed to the surface of a resonantly excited plasmonic nanostructure. For this two‐reagent aqueous synthesis, the surfactant Brij S100 performs the dual roles of reducing and shape‐directing agent while HAuCl_4_ serves as the Au precursor. Herein, it is demonstrated that this photochemical pathway can be transformed into a surfactant‐free nanoplate synthesis through the mere substitution of Brij S100 with a monoalcohol, all while preserving yield and shape control. Mechanistic investigations guided by density functional theory (DFT) calculations indicate the {111} surface‐selective co‐adsorption of monoalcohols in the presence of adsorbed Cl leads to two‐dimensional growth. The work, hence, demonstrates that shape control is achievable in a monoalcohol‐directed synthesis, provides a surfactant‐free approach for obtaining substrate‐based Au nanoplates, and furthers the understanding of the photochemistry needed to achieve these outcomes.

## 2 Results and Discussion

It is widely accepted that the growth of Au nanoplates in liquid media is reliant on the formation of seeds lined with stacking fault defects along a single <111> direction [30, 31]. In a prior study, we demonstrated that such seeds can be formed in periodic arrays through a procedure that uses nanoimprint lithography in combination with a high‐temperature vapor‐phase assembly process [39]. These seeds, which have been employed in a wide variety of nanoplate syntheses [39, 40, 41, 42, 43, 44], are exclusively used in this investigation. It is noteworthy that such seeds, because they are formed in the vapor phase, enter the nanoplate syntheses without ever being exposed to a capping or stabilizing agent. In the current study, syntheses were carried out at room temperature by immersing a seed array in a continuously stirred growth solution of HAuCl_4_, H_2_O, and a monoalcohol (*i.e*., MeOH, EtOH, i‐PrOH) where reactant concentrations and lighting conditions were systematically varied (see Supporting Information for all experimental procedures). Nanoplate growth can be terminated at any point in the reaction by removing the seed array from the growth solution and restarted through its reinsertion. This removal−reinsertion process, which has been used in prior studies [41, 44], allows for the spectroscopic monitoring of the Au plasmon for a single sample at various time‐points in the reaction. It should be noted that control experiments confirm that this procedure does not adversely alter nanoplate growth.

Before presenting the results, it is instructive to provide an understanding of how these substrate‐based nanoplate syntheses differ from the aforementioned surfactant‐free monoalcohol‐based colloidal syntheses where shape control is absent. The foremost difference is that the nanoplate synthesis is seed‐mediated whereas the colloidal synthesis relies on seed formation derived from a LaMer‐like burst nucleation event [26, 45]. As such, nanoplate syntheses proceed through the heterogeneous deposition of reduced Au onto seeds specifically designed to facilitate planar growth. The synthesis parameters are, hence, chosen to promote slow growth while operating in a regime where spontaneous seed formation is improbable. In contrast, burst nucleation requires the rapid supersaturation of the growth solution with reduced Au species so that a large population of clusters can, in a near‐simultaneous manner, exceed their critical radius and then continue to grow at a more moderate rate. As a consequence, the nanoplate growth solution is acidic whereas the colloid is formed under alkaline conditions since a base must be added to the growth solution to increase the concentration of alkoxides (*i.e*., the reducing agent) to the point where Au supersaturation can be achieved through the rapid injection of highly concentrated HAuCl_4_ [26]. Also significant is that the number of substrate‐based seeds added to the growth solution is on the order of 10^8^, a value that is several orders of magnitude smaller than that typically present in a colloidal synthesis. Additionally, growing colloids have translational and rotational degrees of freedom whereas substrate‐based structures are firmly affixed to their support where there is the potential for their growth to be influenced by the underlying substrate material. Seed attachment to the substrate also negates the need for a stabilizing agent, contrasting sharply with colloidal syntheses where achieving stable nanoparticles is a primary consideration. Taken together, it should be recognized that the substrate‐based syntheses are related to, but distinct from, their colloidal counterparts.

**FIGURE 1:**
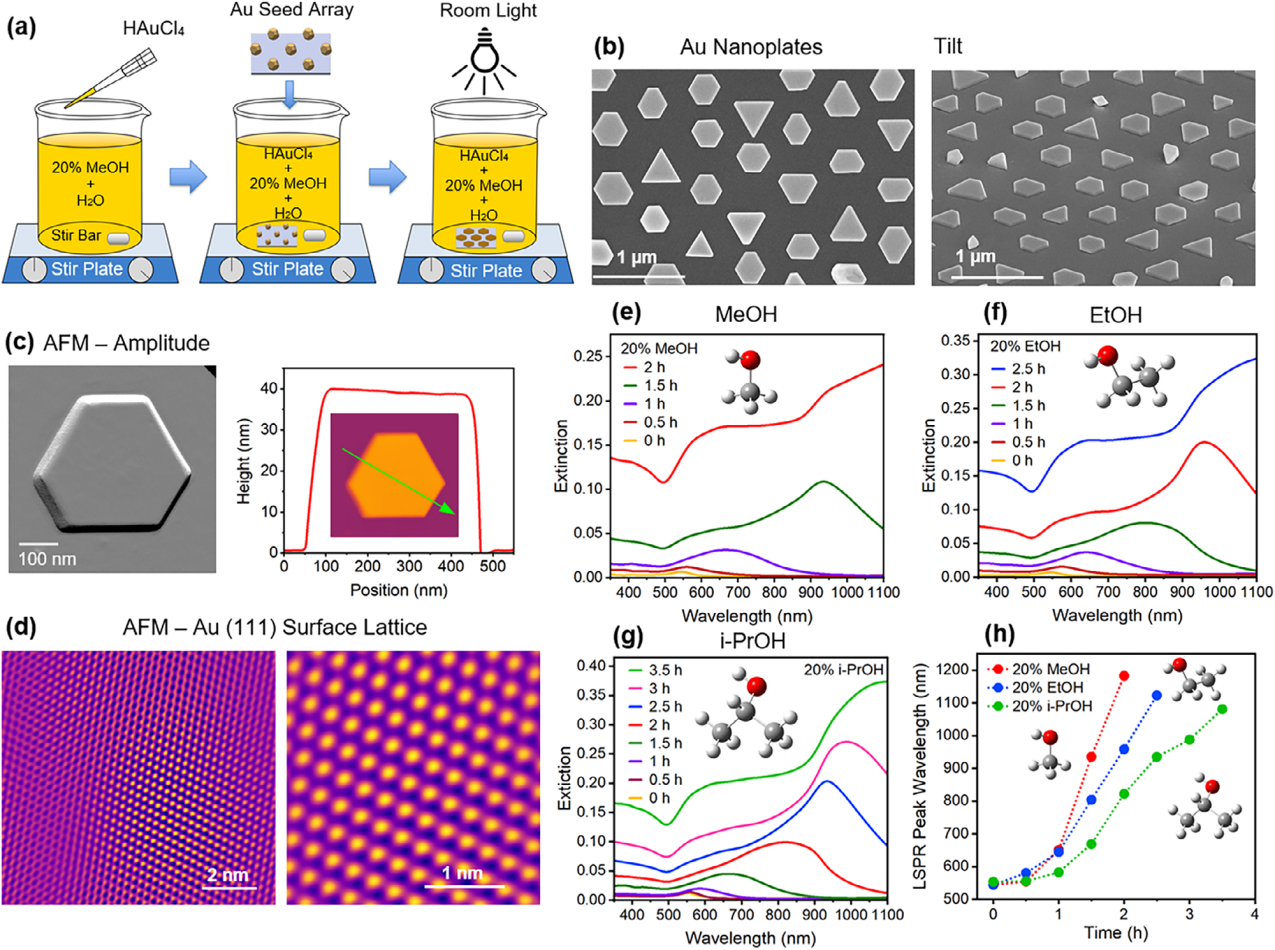
(a) Schematic illustration of a nanoplate synthesis carried out using a 20% MeOH growth solution. (b) Top‐ and tilted‐view SEM images of the nanoplate array. (c) AFM amplitude image and the associated line scan for a Au nanoplate. (d) FFT autocorrelated atomic stick‐slip image of the (111) Au nanoplate surface lattice. Time progression in the extinction spectra as seeds grow into nanoplates for (e) MeOH‐, (f) EtOH‐, and (g) i‐PrOH‐based growth solutions. (h) Time‐dependence of the LSPR peak wavelength for nanoplate arrays grown from the various growth solutions.

Figure 1a shows a schematic illustrating a nanoplate synthesis carried out under room light using a 20% v/v MeOH growth solution that proceeds for 2 h before the sample is withdrawn. SEM images of the structures formed (Figure 1b; Figure S1) reveal nanoplates arranged in periodic arrays along with some unwanted three‐dimensional structures. These unwanted structures, which have also been observed in our surfactant‐based nanoplate syntheses at similar yields, originate from seeds that lack the internal defect structure needed to promote planar growth [39]. The nanoplates are highly faceted with shapes that are either hexagonal or triangular. The alignment of the side‐faceting on adjacent structures is a consequence of the heteroepitaxial relationship formed between the seed and the underlying [0001]‐oriented sapphire substrate [46]. AFM characterization reveals nanoplate thicknesses of approximately 38 nm (Figure 1c). Nanoplates formed using growth solutions where the MeOH is replaced by an identical volume of EtOH or i‐PrOH exhibit similar characteristics (Figures S2 and S3). Moreover, all of these nanoplate arrays, from a morphological perspective, are near‐identical to those formed when using surfactants [39, 41]. The key difference, however, is that the nanoplates, once removed from the growth solution and rinsed in common solvents, express a surface where the (111) close‐packed atoms of Au are clearly visible in an AFM FFT autocorrelated atomic stick‐slip image (Figure 1d). In sharp contrast, no such surface lattice image is obtainable for similarly prepared nanoplates when grown using the Brij S100 surfactant. As a further demonstration of surface quality, surfactant‐free and surfactant‐grown nanoplates were assessed as cores in a core–satellite assembly procedure. This seemingly straightforward assembly process sees the functionalization of the core with a C8DT dithiol linker (i.e., 1,8‐octanedithiol) followed by its exposure to a colloid of much smaller nanoparticles (i.e., satellites). Successful assembly, which is characterized by the dense attachment of satellites to the core, is, however, highly sensitive to the surface quality of the core [47]. SEM images reveal that the satellites assembled onto the functionalized surfactant‐free nanoplate surface at high density, whereas the surfactant‐grown structures displayed only sparse coverage due to their surfactant‐corrupted surface (Figure S4). These results provide an initial indication that there is significant benefit derived from the use of this monoalcohol‐based growth mode.

Nanoplate syntheses were carried out using growth solutions at the 20% concentration for MeOH, EtOH, and i‐PrOH while spectroscopically characterizing the plasmon of the emerging structures at half‐hour intervals for wavelengths extending from 350 to 1100 nm (Figure 1e–g). Spectra obtained at the conclusion of each of the three syntheses for wavelengths up to 2500 nm are provided in Figure S5. For all three cases, the data show a time progression in which (i) a localized surface plasmon resonance (LSPR) grows in magnitude as it redshifts to higher wavelengths and (ii) the emergence of a shoulder near 600 nm. The red‐shifting LSPR is attributable to the plasmon of the emerging nanoplates, whereas the shoulder arises from the growth of the unwanted three‐dimensional structures [44]. Extracted from this data, and shown in Figure 1h, is the time dependence of the LSPR peak position for each of the three monoalcohol‐based syntheses. It has been demonstrated both experimentally and theoretically that plots of the LSPR peak position vs. growth time and the nanoplate edge‐length vs. growth time follow similar trendlines [39, 41, 48, 49]. As such, the LSPR time dependence acts as a surrogate for monitoring increases to the nanoplate size where the slope of the curve provides a measure of the growth rate. With this understanding, it is evident from Figure 1h that the MeOH‐based synthesis leads to the fastest growth whereas the i‐PrOH synthesis is the slowest. It is noteworthy that this MeOH>EtOH>i‐PrOH progression is in accordance with the inverse 15.5<15.9<17.1 progression in the pKa values for the monoalcohols. Given that lower pKa values correspond to a greater ease by which deprotonated species are formed, the observed trends imply that alkoxide formation is preferred in the faster‐growing growth solutions. It should, however, be recognized that the comparisons of MeOH, EtOH, and i‐PrOH are based on equal monoalcohol‐to‐H_2_O volume ratios but the molarities are far from equal (Table S1) and this influence also favors the same progression. Other factors such as viscosity, which follow a similar trend (Table S2), can also play a role in establishing the growth rate. Also apparent from the data is that growth proceeds slowly in the early stages of the reaction followed by an upturn in the growth rate (*i.e*., a gradual slope followed by an upturn), a behavior that is exaggerated for i‐PrOH.

**FIGURE 2:**
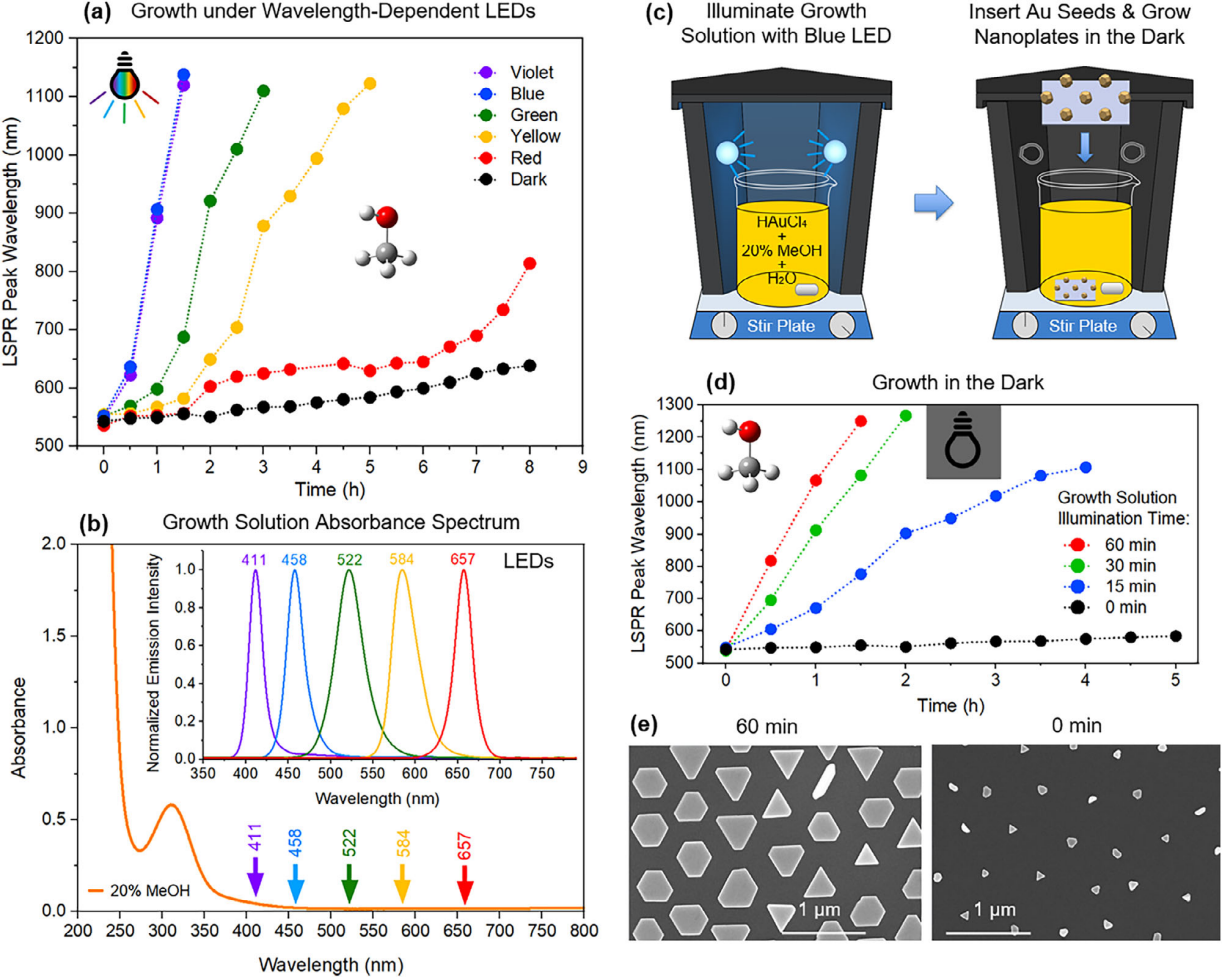
(a) Dependence of the LSPR peak position on growth time for nanoplates grown under wavelength‐dependent LED illumination. (b) Absorbance spectrum of a growth solution containing 20% MeOH where the inset displays the normalized emission intensity for each of the LED wavelengths used. (c) Schematic illustration of a synthesis where the growth solution is first illuminated, after which the light is extinguished and the substrate‐based seeds are inserted and grown in the dark. (d) LSPR peak position vs. growth time for various growth solution illumination times and (e) nanoplate SEM images for growth solution illumination times of 60 and 0 min.

Wavelength‐dependent syntheses were used to establish the role of light in driving the growth of nanoplates. For these experiments, samples were grown in much the same manner as shown in Figure 1a except that the beaker containing the growth solution was placed in a 3D‐printed opaque housing equipped with three interchangeable light‐emitting diodes (LEDs), selected from a set of five peak emission wavelengths: 411 (violet), 458 (blue), 522 (green), 584 (yellow), and 657 nm (red). The light intensity was set to 1 W m^−2^ because this low light level allowed for single‐day data acquisition at all wavelengths while maintaining intensities that do not lead to significant heating even when the nanostructures are resonantly excited [50, 51, 52]. For these syntheses, a growth solution with a 20% MeOH concentration was utilized. Figure 2a shows the time dependence of the LSPR peak position for nanoplates grown at each of the excitation wavelengths as well as for a synthesis carried out in the dark. The data reveal that the growth rate is largest under blue and violet light, followed by a continuous decline as the wavelength is increased. The sample prepared in the dark shows little growth even after 8 h, a clear indication that the synthesis is light‐driven. The dataset closely follows the trends exhibited in our prior study [41] where identical experiments were carried out using a growth solution that substituted the MeOH component for the Brij S100 surfactant. In both cases, the behavior is inconsistent with a plasmon‐mediated growth mode since the highest growth rates occur for wavelengths well below the plasmon resonance of the seeds (*i.e*., 550 nm) and where no enhancements to the growth rate are observed at longer wavelengths when the seeds or emerging nanoplates are in resonance with the incident light.

Given the similar behaviors observed for both the surfactant (*i.e*., Brij S100) and surfactant‐free syntheses (*i.e*., MeOH), it was postulated that both syntheses follow the same mechanistic pathway where, for the surfactant case, it is established that photoexcitations within the growth solution are the key driver of nanoplate growth [41]. In an effort to establish whether this is indeed the case, the absorbance spectrum of the surfactant‐free growth solution was first compared to the spectral outputs of the various LEDs (Figure 2b). The data reveal that spectral overlap occurs between the tail of the growth solution absorbance peak centered at 310 nm and the outputs of both the blue and violet LEDs. This absorbance peak is, to a large degree, associated with a ligand‐to‐metal charge transfer within the AuCl_4_^−^ ion [53, 54], however, the presence of methanol results in both a slight intensification of the peak and a 5 nm redshift. The alterations caused by MeOH likely relate to a shift in the polarity of the solvent [55]. A complete spectral analysis of all components within the growth solution is presented in Figure S6. It is also shown that the MeOH‐, EtOH‐, and i‐PrOH‐based growth solutions exhibit near‐identical absorbance spectra (Figure S7). A seeming inconsistency with assigning the light‐driven nature of the growth mode to excitations within the growth solution is that nanoplates grown under violet light show a near‐identical growth rate to those grown under blue light (Figure 2a) despite a larger overlap between the emission spectrum of the violet LED and the absorbance spectrum of the growth solution (Figure 2b). Such behavior is likely attributable to the rate‐limiting step in the growth rate becoming a factor other than the photon dose applied. A possible rate‐limiting step is the rate at which reduced Au is able to attach to the side‐facets of an emerging nanoplate in the layer‐by‐layer manner needed to maintain crisp faceting. It should also be noted that the behaviors described as they relate to violet and blue light are also observed in the Brij S100‐based growth [41].

The use of substrate‐bound seeds allows for a decisive test that is able to determine whether photoexcitations within the growth solution are the primary driver of the redox chemistry needed to induce nanoplate growth. The test, which is shown schematically in Figure 2c, sees the growth solution illuminated with blue light for times as long as 60 min, after which the substrate‐bound seeds are immersed and allowed to grow in the dark where their repeated removal and reinsertion allow for spectroscopic monitoring of the emerging structures. The experimental design, hence, decouples the photochemical and growth steps such that the seeds and emerging structures never undergo any illumination. Therefore, if the structures require illumination for growth to proceed, then no growth is expected; however, if the required photochemistry occurs within the growth solution, then nanoplates will emerge. Figure 2d shows a comparison of the time dependence of the LSPR peak position for growth solutions that underwent illumination times of 0, 15, 30, and 60 min. It reveals that nanoplate growth ensues only for the cases where the growth solution was “optically primed” prior to seed insertion, with longer durations under blue light giving rise to faster growth rates. Post‐synthesis SEM images confirm that nanoplates do indeed form under these growth conditions (Figure 2e; Figure S8). It is noteworthy that nanoplate growth for the 60 min optically primed case is slightly faster than that observed when growth solution illumination commences at the same time as seed insertion (Figure S9). The faster rate is likely attributable to the absence of a time lag between the onset of illumination and the build‐up of sufficient quantities of reduced Au within the growth solution. Consistent with the formation of reduced Au is a gradual reduction in the AuCl_4_^−^ absorbance peak of the growth solution as the illumination time is increased (Figure S10). Taken together, these data firmly establish the monoalcohol‐based synthesis as only the second light‐mediated nanoplate growth mode to proceed along this alternate mechanistic pathway.

**FIGURE 3:**
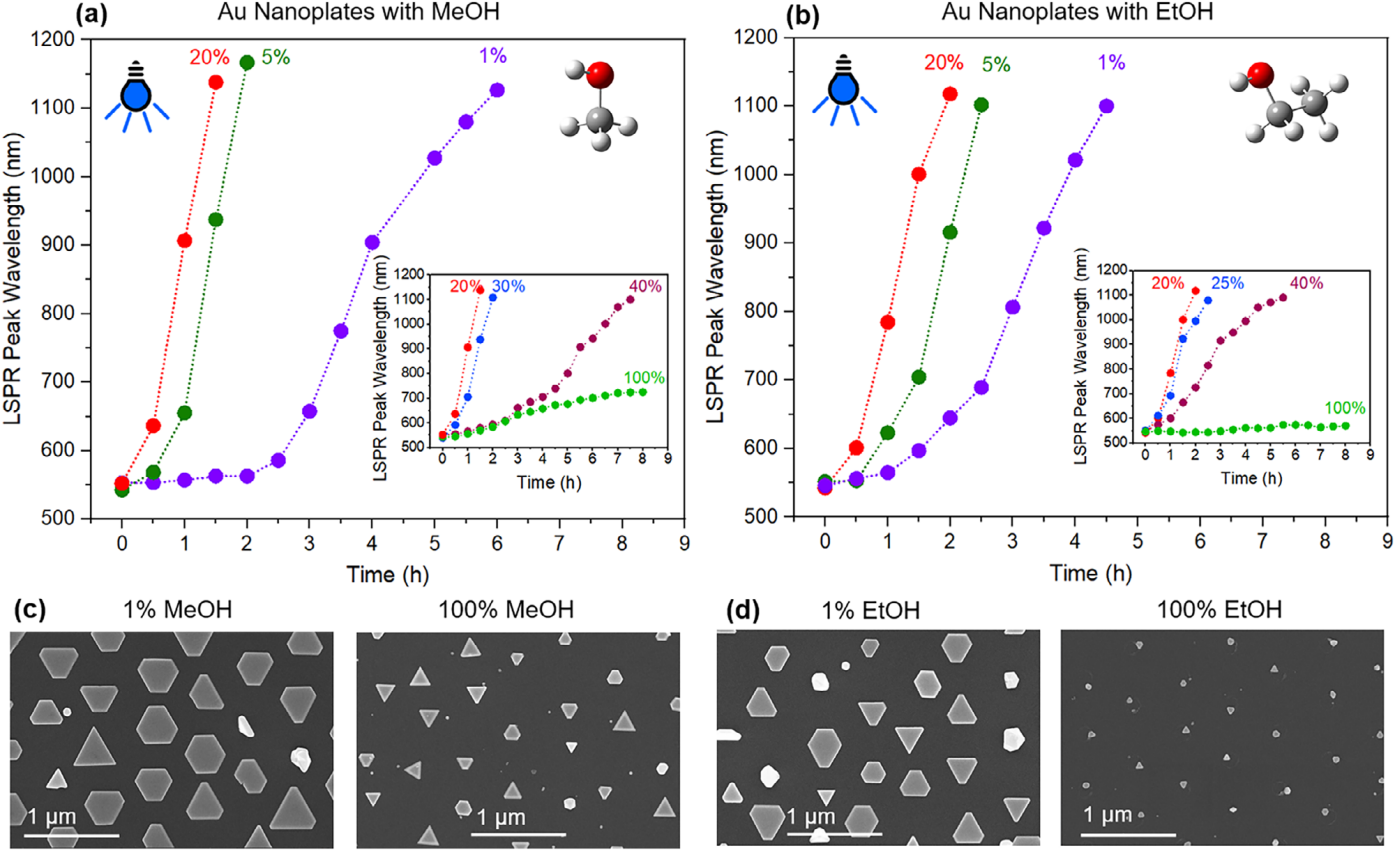
Dependence of the LSPR peak position on growth time for nanoplates grown using growth solutions with varying percentages of (a) MeOH and (b) EtOH. SEM images of nanoplates grown using 1% and 100% (c) MeOH and (d) EtOH.

The strong dependence on the monoalcohol concentration observed for the surfactant‐free colloidal synthesis [7, 26, 29] motivated a similar evaluation of this parameter for the seed‐mediated substrate‐based growth under 1 W m^−2^ blue light illumination. Figure 3 shows the time‐dependence of the LSPR peak position for MeOH and EtOH concentrations ranging from 1% to 100% as well as SEM images of the nanoplates formed at the lowest and highest concentrations. For both cases, the reaction proceeds at a progressively faster rate up to a concentration of approximately 20%, followed by a fairly rapid decline (see insets to Figure 3a,b). In contrast to the colloidal investigations [7, 26, 29], growth occurs even for 100% MeOH and EtOH, albeit at an exceedingly slow rate. Even though the HAuCl_4_ was dissolved in the monoalcohol for these experiments, trace levels of H_2_O in both the alcohol and HAuCl_4_·3H_2_O powder cannot be ruled out as an initiator for this low‐level growth. Moreover, it should be recognized that if similar quantities of Au were to be reduced in solution for the colloidal and substrate‐based cases, the seeds would provide an opportunity for heterogeneous deposition whereas, for the colloidal case, there may not be enough reduced Au within the growth solution to form particles at sizes exceeding the critical radius. It should also be recognized that the slow growth at these higher concentrations is not associated with a reduced absorbance in the growth solution since measurements taken over the entire 0% to 100% range reveal an absorbance that, in general, intensifies and redshifts as the monoalcohol concentration is increased (Figure S11).

One of the striking features of the data in Figure 3a,b is that the slopes of the curves do not vary widely in the final stages of the reaction for MeOH and EtOH concentrations in the range of 1% to 30%. Instead, the major difference between the curves is the time required before the onset of this slope. With the slope being an indicator of growth rate, the data point toward a synthesis that is sluggish in its early stages for suboptimal monoalcohol concentrations but then becomes quite similar in the later stages of the reaction. The similar slopes are, once again, consistent with a rate‐limiting effect on the growth rate being something other than the applied photon dose. It is tempting to attribute the early‐stage sluggishness to a gradual buildup of the alkoxide reducing agent that, in turn, gives rise to a gradual buildup of reduced Au; however, this does not account for the fact that the upturns in the slope appear abrupt. A possible explanation is that the early stages of the reaction see a gradual buildup of reduced Au that, in time, leads to homogeneous nucleation of a small population of colloidal nanostructures that act as catalysts capable of accelerating the ongoing redox reaction. Although this postulate is admittedly speculative, it has been verified that a low‐concentration colloid emerges within the growth solution that will, given enough time, grow to large sizes as it consumes the available reactants (Figure S12). It should also be noted that the existence of autocatalytic growth modes is well‐accepted [25, 45] and that sluggish growth followed by a rapid acceleration has been reported for both monoalcohol‐ [26, 29, 56] and polyol‐based syntheses [57].

**FIGURE 4:**
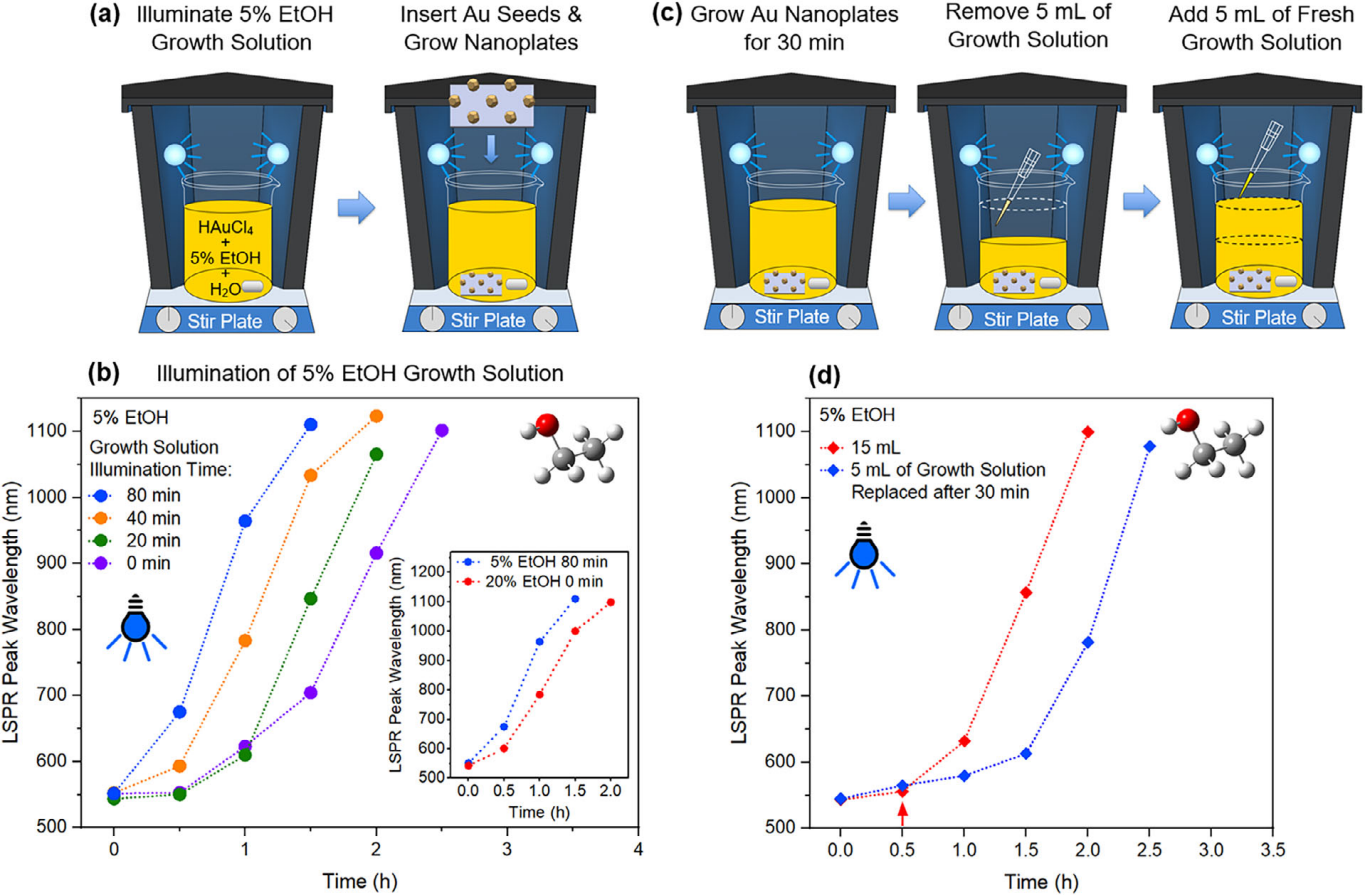
(a) Schematic illustrating syntheses where a suboptimal 5% EtOH growth solution is illuminated with blue light for intervals in the range of 0 to 80 min followed by the insertion of substrate‐based seeds and (b) the associated data showing the dependence of the LSPR peak position on growth time where the inset compares the 80 min response to that grown at the optimal 20% EtOH concentration using standard growth conditions. (c) Schematic illustrating a synthesis where a seed array is immersed into a 15 mL 5% MeOH growth solution followed by its illumination with blue light for 30 min after which 5 mL of the growth solution is removed and replaced with an equivalent amount of fresh solution and (d) the associated data showing the dependence of the LSPR peak position on growth time.

Two additional syntheses were designed to provide supportive evidence showing both the ability to manipulate the onset of rapid nanoplate growth and the existence of a rate‐limiting step in the growth rate. These syntheses were intentionally carried out using a suboptimal 5% EtOH solution under 1 W m^−2^ blue light. In the first synthesis, the growth solution is illuminated for periods ranging from 0 to 80 min prior to seed insertion, after which nanoplate growth is spectroscopically monitored under continued blue light exposure (Figure 4a). The experimental design, hence, allows the photochemistry to proceed for various time intervals before the substrate‐based nanoplate growth is initiated. Figure 4b shows the time‐dependence of the LSPR peak position for the four growth solution illumination times studied. The data indicate an immediate onset of nanoplate growth for the growth solution that was illuminated for 80 min where each successive reduction in illumination time leads to a longer delay before a near‐identical growth rate is achieved. Another important observation is that substrate‐based nanoplate growth for the 80 min pre‐illumination of the suboptimal 5% EtOH growth solution can proceed at a rate that exceeds that of an optimized 20% EtOH solution grown under standard conditions (inset to Figure 4b). The mechanistic implication of the result is that the necessary redox chemistry is occurring in the 5% EtOH growth solution at a slower rate, but this deficiency in rate can be compensated for through the pre‐illumination of the growth solution. In the second synthesis, nanoplates are allowed to grow in a 15 mL growth solution for 30 min at which point 5.0‐mL quantities of growth solution are removed and equivalent amounts of fresh solution are added (Figure 4c). The experimental design, hence, leads to a partial removal of the photochemical reaction products and the dilution of what remains while keeping the total volume constant. The time‐dependence of the LSPR peak position, once again, shows a growth rate that is near‐identical in the latter stages of the reaction but where the onset of this rate is progressively delayed for higher dilutions. Taken together, this data shows consistency in behavior even when synthetic procedures are significantly altered.

To elucidate the role of monoalcohol‐derived species in directing Au nanoplate growth, DFT calculations were employed. Prior theoretical studies have shown that highly anisotropic kinetically‐grown nanostructures, such as nanowires and nanoplates, can have an underlying thermodynamic driving force, such as the minimization of surface energy [58, 59]. The most obvious choice for solution‐phase species that could drive nanoplate growth are monoalcohols and chloride. Previous work has shown that synergy in the adsorption of organics and halides can lead to shape‐selective growth of metal nanostructures [58, 59, 60, 61, 62, 63].

Ab initio thermodynamics was used to calculate the surface energies of Au(100) and Au(111) in the presence of adsorbed MeOH and Cl. The surface energy γ_*i*_ of surface *i* was calculated using

(1) $$\gamma_{i}=\frac{E_{slabi}-N_{Au}\mu_{Au}-N_{MeOH}\mu_{MeOH}-N_{Cl}\mu_{Cl^{-}}}{A_{slab}}-\frac{E_{fixed}-N_{Au}\mu_{Au}}{2A_{slab}}$$

where *E*_*slab* *i*_ is the energy of the Au slab *i* [*i* = (100) or (111)] with adsorbed species, *N_Au_* is the number of Au atoms in the slab, and µ_*Au*_ is the bulk energy of Au. *N_MeOH_* is the number of adsorbed MeOH and µ_*MeOH*_ is the solution‐phase chemical potential of MeOH. *N_Cl_* is the number of adsorbed Cl and $\mu_{Cl^{-}}$ is the chemical potential of solution‐phase chloride. In these calculations, the chloride ion (Cl^−^) is considered to reside in the solution phase, but neutral Cl is adsorbed on the surface. The solution‐phase chemical potentials are parameters that are varied to understand how surface composition changes with solution‐phase concentrations, as the chemical potential is proportional to concentration. Because a slab is used with adsorbed species and relaxation on one side only, there is a need to account for the asymmetry in surface energy. *E_fixed_* is the energy of a Au slab with all atoms fixed to the bulk positions and *A_slab_* is the area of the surface.

**FIGURE 5:**
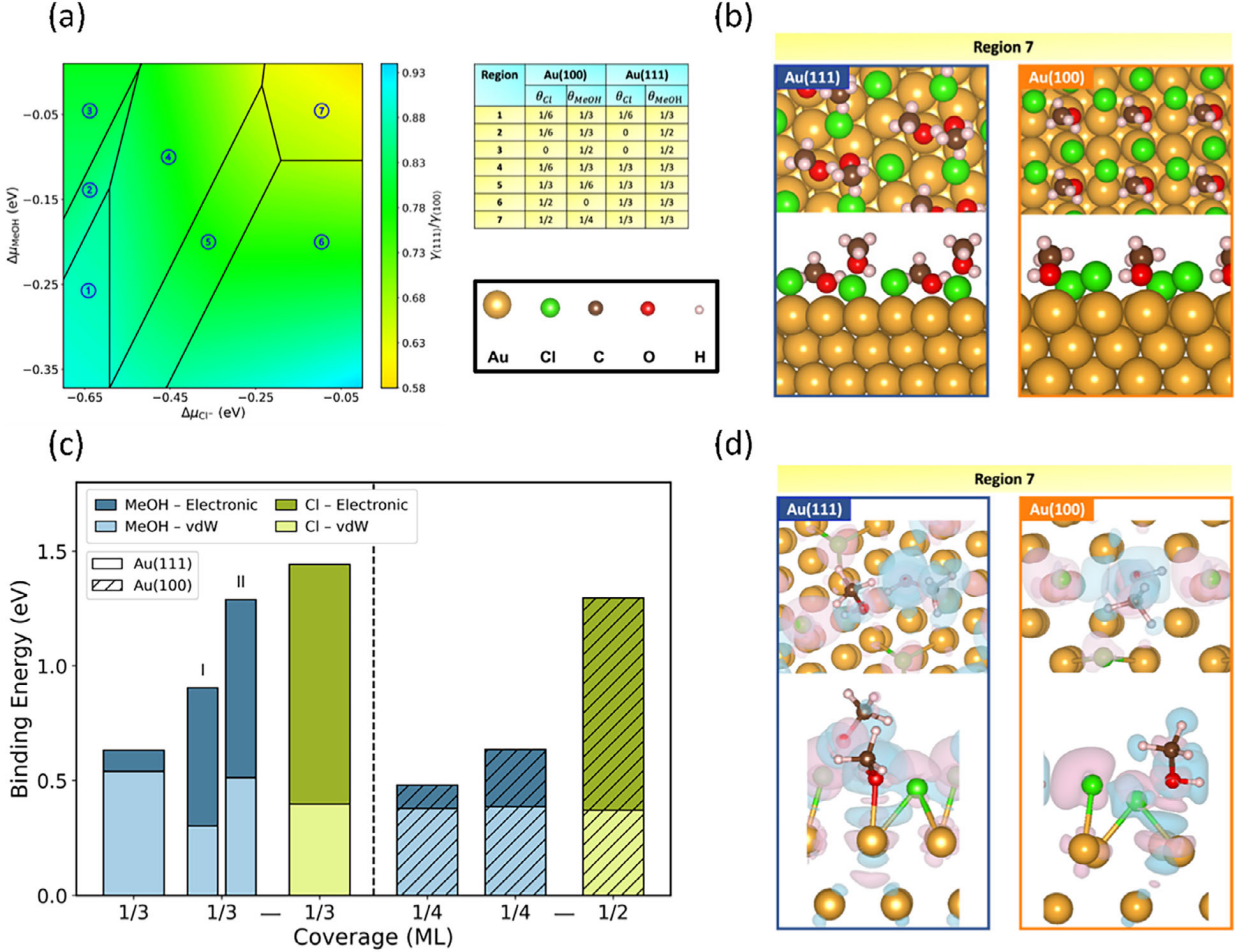
(a) Phase diagram showing the surface energy ratio γ_111_/γ_100_ as a function of solution‐phase MeOH and Cl^−^ chemical potentials, Δµ_*MeOH*_ and $\Delta \mu_{Cl^{-}}$, respectively, referenced to the gas‐phase values. The table indicates the adsorbate coverages *θ_Cl_* and *θ_MeOH_* (in ML) for various regions of the diagram. (b) Configurations for Region 7 on Au(111) and Au(100). (c) Binding energies on Au(111): adsorbed MeOH at θ_*MeOH*_ = 1/3, the binding energy of each of the two MeOH (I is farther from the Au surface and II is closer) in the unit cell in Region 7 (θ_*MeOH*_ = 1/3), and the binding energy of Cl in Region 7 (θ_*Cl*_ = 1/3). Binding energies on Au(100): adsorbed MeOH at θ_*MeOH*_ = 1/4, the binding energy of MeOH in the unit cell in Region 7 (θ_*MeOH*_ = 1/4), and the binding energy of Cl in Region 7 (θ_*Cl*_ = 1/2). The total binding energy is delineated as the sum of vdW and electronic interactions. (d) Electron density difference induced by adsorbed MeOH, given by Equation (2), for Au(111) and Au(100) in Region 7. The iso‐surface level is 0.001 e/Å^3^ for both systems and pink indicates charge accumulation (increase in electron density), while blue indicates charge depletion (decrease in electron density).

From the surface energies of 117 Au(100) and 115 Au(111) facets with various fractional coverages of MeOH and Cl, a phase diagram was created to show the most likely surface configurations (i.e., those with minimum energy) as a function of the chemical potentials of solution‐phase MeOH and Cl^−^. In all, 23 different regions were identified, as shown in Figure S13. Figure 5a shows a subset of the full phase diagram of the Au(100) and Au(111) surfaces deemed most relevant for Au nanoplate growth as a function of the solution‐phase MeOH and chloride chemical potentials Δµ_*MeOH*_ and $\Delta \mu_{Cl^{-}}$, respectively, referenced to the gas‐phase values. As indicated by the color scale, the surface‐energy ratio γ_111_/γ_100_ decreases significantly with increasing MeOH and Cl^−^ concentration. Region 7 is likely the most relevant to experiment. Here, γ_111_/γ_100_ ≈ 0.6, which is significantly lower than our calculated bare‐surface ratio of γ_111_/γ_100_ = 0.91 and lower than that found for the growth of Cu microplates in the presence of iodine and hexadecylamine [58]. Such a low surface‐energy ratio provides a strong driving force for nanoplate growth.

Figure 5b shows the surface configurations for both facets in Region 7 of Figure 5a. In Region 7, Au(100) has θ_*Cl*_ = 1/2 ML and θ_*MeOH*_ = 1/4 ML, while Au(111) has θ_*Cl*_ = θ_*MeOH*_ = 1/3 ML. Here, one monolayer (ML) corresponds to one adsorbate for every surface atom. To analyze the origins of the surface‐energy lowering effect of co‐adsorbed Cl and MeOH, several different calculations were performed. First, the binding energies were calculated for MeOH and Cl in the surface configurations in Figure 5b. These are shown in Figure 5c, where it is seen that both MeOH and Cl bind stronger to Au(111) than to Au(100). Since the capability of an adsorbed species to lower the surface energy depends on its binding strength [64], this is an expected result that aligns with the surface‐energy ratio for Region 7 in Figure 5a. It is also evident in Figure 5c that the binding energy is higher for MeOH when it is co‐adsorbed with Cl than when it is adsorbed without Cl at the same coverage. The binding energies in Figure 5c are partitioned into van der Waals (vdW) and direct‐bonding, electronic interactions, which indicates that both adsorbed MeOH in the unit cell on Au(111) for Region 7 (Figure 5b) interact primarily with the surface via direct‐bonding electronic interactions. This is interesting, in that the distance between the O in MeOH I and the closest Au surface atom is 3.80 Å, though the distance to the closest Cl atom is 3.07 Å. In contrast, the binding energy for MeOH adsorption on Au(111) in the absence of adsorbed Cl is primarily due to vdW interactions. For Au(100), the electronic component of the binding energy increases for MeOH‐Cl co‐adsorption, but the vdW component remains the same as for MeOH in the absence of adsorbed Cl.

The charge‐density difference induced by MeOH adsorption on the two Cl‐covered Au surfaces in Region 7 was also calculated. This charge‐density difference is given by

(2) $$\Delta \rho_{MeOH}=\rho_{slabi}-\rho_{MeOH}-\rho_{Au+Clslabi}$$

where ρ_*slab* *i*_ is the electron density of Au slab *i* [*i* = (100) or (111)] with adsorbed MeOH and Cl, ρ_*MeOH*_ is the electron density of the MeOH adsorbate(s) with the same configuration as on the slab, but without the slab present, and ρ_*Au* + *Cl* *slab* *i*_ is the electron density of Au slab *i* with adsorbed Cl in the configurations for Region 7.

As seen in Figure 5d, the adsorption of MeOH induces significant charge redistribution to the Cl‐covered Au surfaces. Studies of the work function change when Cl adsorbs on Au(111) indicate that Cl acquires charge from the surface [65, 66]. Figure 5d (especially the top‐down views) shows that in the presence of adsorbed MeOH, additional charge is transferred from the substrate to the Cl atoms (pink iso‐surfaces) on both surfaces. It is also evident that there is a complex charge redistribution within adsorbed MeOH that underlies the increase in the electronic interaction from a surface with no adsorbed Cl to co‐adsorption of MeOH and Cl in Figure 5d. It is seen that MeOH I, which is the furthest away from Au(111), has significant interaction with adsorbed Cl through its O atom. Figure 5d shows, particularly from the side view, that MeOH is polarized by its interaction with Au(100), with a build‐up of charge density near the adsorbed Cl and a depletion of charge density (blue iso‐surfaces) around the H atoms. Thus, DFT calculations show that a facet‐selective synergy in the binding of Cl and MeOH to Au surfaces could provide a strong driving force for nanoplate growth by reducing the surface‐energy ratio γ_111_/γ_100_ significantly below the value for the bare surfaces. Since MeOH exhibits similar binding trends to the other monoalcohols in these studies (see Figure S14), it seems likely that this is a general mechanism.

It is worth mentioning that the influence of methoxide (MeO) on the surface‐energy trends of the Au surfaces was also considered, as it is known that Au can catalyze the decomposition of MeOH via the reaction [67, 68, 69, 70]

$$CH_{3}\mathrm{OH}\to CH_{3}O+H$$

or

$$CH_{3}\mathrm{OH}+\mathrm{Cl}\to CH_{3}O+\mathrm{HCl}$$

in the presence of adsorbed Cl. Because of its instability in the gas phase, MeO binds strongly to the Au surfaces relative to MeOH (see Figures S14 and S15), but it binds more strongly to Au(100) than to Au(111). For MeO to be a major species driving nanoplate growth, it needs to be prevalent on Au(111), but not Au(100). This could occur due to kinetics if MeOH decomposition is more facile on Au(111) than on Au(100). To assess this possibility, climbing‐image nudged elastic band [71] (CI‐NEB) calculations were performed to determine the energy barrier for the decomposition of MeOH to MeO on both facets and in the absence and presence of adsorbed Cl. In these calculations, the MeOH coverage was fixed at 1/6 ML.

**FIGURE 6:**
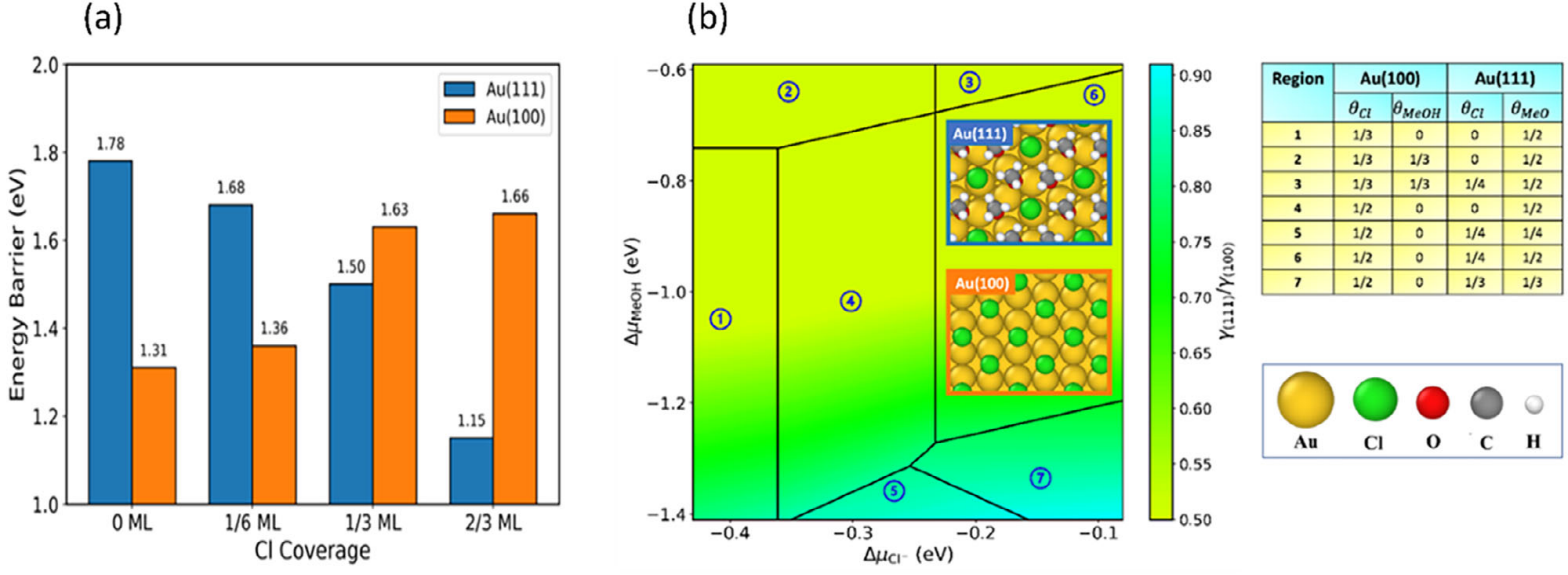
(a) Energy barrier for MeOH‐MEO conversion on Au(111) (blue) and Au(100) (orange) as a function of Cl coverage at a fixed MeOH coverage of 1/6 ML. (b) Phase diagram showing the surface energy ratio γ_111_/γ_100_ as a function of the MeOH and Cl^−^ chemical potentials, referenced to gas‐phase values. Insets show the configurations with the lowest surface energy in Region 6 on Au(111) and Au(100). The table presents the adsorbate coverages *θ* in various regions of the phase diagram.

Figure 6a summarizes the calculated energy barriers for each Cl coverage. The energy barrier for MeOH decomposition on bare Au(111) is 1.78 eV and this decreases to 1.15 eV as θ_*Cl*_ increases. It is noted that the high energy barrier on bare Au(111) is consistent with a previous theoretical investigation [68]. Conversely, on Au(100), the barrier increases from 1.31 eV on the bare surface to 1.66 eV as θ_*Cl*_ increases. There is a cross‐over in the (111)‐(100) energy barriers between θ_*Cl*_ = 1/6–1/3 ML: below this cross‐over coverage, MeOH decomposition is more facile on Au(100) and above it, MeOH decomposition is easier on Au(111). Thus, these calculations indicate that in the presence of adsorbed Cl, relevant to the experimental synthesis, MeOH activation is favored on Au(111) relative to Au(100). This could mean that MeO preferentially accumulates on Au(111), whereas MeOH remains predominantly in its molecular form on Au(100). As shown in Figure 6b, this scenario could also provide a strong driving force for nanoplate growth in Region 6 where γ_111_/γ_100_ is as low as 0.5. Here, we express the chemical potential of MeO in terms of the chemical potential of MeOH using $\mu_{MeO}=\mu_{MeOH}+\mu_{MeO}^{gas}-\mu_{MeOH}^{gas}$, where the superscript “gas” refers to the gas‐phase chemical potentials of these two species. A caveat to these results is that the reverse barrier for MeO and HCl to transform to MeOH and Cl is significantly smaller than the forward barrier. However, there are aspects of these surfaces that we did not probe, including the possibility that MeOH and MeO are co‐adsorbed. Thus, we keep this as a possibility, but following Occam's razor principle, the co‐adsorption of MeOH and Cl, and its effect of lowering γ_111_/γ_100_ significantly below the value for the bare surfaces, appears to be the simplest explanation for nanoplate growth.

The synthesis of faceted noble metal nanostructures in the absence of surfactants is by no means a new discovery [72]. Capping‐agent‐free approaches have, for example, been demonstrated where nanostructures are formed on supports to mitigate the need for a stabilizing agent [73−75]. Additionally, high‐temperature vapor‐phase methods have been widely used to obtain substrate‐supported nanometals where faceting originates from the structures assuming either thermodynamically‐ or kinetically‐favored configurations [76−78] The synthesis demonstrated in the current report, however, distinguishes itself from these examples in that it is an additive‐free, monoalcohol‐based, room‐temperature, liquid‐phase growth pathway for obtaining periodic arrays of highly‐faceted nanoplates. Unlike surfactant‐free monoalcohol‐based colloidal syntheses, where the benefits are typically linked to obtaining nanomaterials with improved catalytic performance [27] or in forwarding sustainable synthetic practices [29], the importance of the current approach resides in the synthesis giving rise to device‐ready nanoplates exhibiting unobstructed planar Au surfaces that do not require any post‐processing cleaning steps directed toward the removal of unwanted surface species. The absence of persistent surface agents negates the possibility of their well‐documented negative influences that include reduced catalytic activity, diminished device performance due to incomplete surface functionalization, a less than optimal adhesion of analytes, uneven and degraded electron‐transfer processes, and reduced biocompatibility [13−19]. Although significant progress has been made in terms of cleaning nanostructure surfaces using chemical and physical methods [14, 15, 17, 79], these approaches are far from ideal. The treatments, which are often tedious and employ hazardous chemicals, can lead to morphological degradation and transformations, incomplete removal of capping agents, unwanted residues and adsorbed species, and additional processing steps that have the potential to diminish reproducibility [47, 80−84]. As such, the synthesis of ready‐to‐use nanoplates with near‐pristine surfaces provides a significant advantage over their surfactant‐based counterparts in terms of advancing substrate‐based nanometal applications [30, 31, 85, 86].

From a mechanistic standpoint, the substrate‐based monoalcohol‐directed Au nanoplate synthesis shares commonalities with monoalcohol‐based colloidal syntheses but also has distinct aspects that allow for shape‐control. In both cases, visible light can be used to trigger the chemical reactions needed to reduce Au. The key difference is that growth solutions in the light‐driven colloidal syntheses [7] are supplemented with NaOH to accelerate the reaction by deprotonating the monoalcohol to increase the supply of the alkoxide reducing agent. The substrate‐based growth, with few seeds and a desire to maintain reduction at low levels to hinder the unwanted spontaneous nucleation of colloidal nanoparticles, would be negatively impacted by a similar acceleration. The observation that both syntheses respond more favorably to high‐energy photons and support growth in a variety of monoalcohols suggests that a common underlying redox chemistry is operative in both systems. Although the details of the photochemical pathway still need to be clarified, the ability to decouple the photochemical and growth steps (see Figure 2c,d) unequivocally demonstrates that the key driver of growth is photoexcitations within the growth solution as opposed to plasmonic excitations within the growing structures. Moreover, the ability to enhance growth rates in the suboptimal 5% ethanol growth solution to levels above the optimal 20% value by illuminating the growth solution prior to seed insertion (see inset to Figure 4b) suggests that the monoalcohol‐to‐H_2_O ratio is largely responsible for determining the reduction rate but, once reduced, the Au is available for deposition over prolonged periods. At the same time, the ability to obtain nanoplates over a wide range of monoalcohol concentrations shows that the ratio does not fundamentally alter the planar growth mode. It is, thus, surmised that the photochemistry in both the colloidal and substrate‐based systems similarly yields solvated neutral Au atoms that are available for deposition onto growing structures but has minimal influence on shape control.

Without any addition of capping agents to the growth solution and existing nanoplate growth modes requiring that such agents be present, the emergence of nanoplates presented somewhat of an enigma. DFT calculations, however, reveal that there is a synergy in the binding of MeOH and Cl that strengthens MeOH binding – particularly on Au(111), effectively stabilizing the {111} facets and directing anisotropic growth. Possible explanations as to why nanoplates are produced in high yield in the substrate‐based synthesis but not in the colloidal syntheses are (i) the self‐nucleation of nanoparticles within colloidal syntheses is not prone to the formation of the stacking fault defects needed to support planar growth modes whereas the substrate‐based growth mode adds seeds specifically designed for this purpose, (ii) an insufficient supply of chlorine to support planar growth in the much larger, and the near‐simultaneously produced, population of colloidal seeds, and (iii) the influence of the sapphire substrate, which blocks growth solution contact with the bottom surface of the nanoplate and significantly alters the local environment in a stirred solution due to the no‐slip condition.

## 3 Conclusion

In summary, a low‐complexity light‐driven synthetic protocol has been demonstrated that is capable of forming arrays of Au nanoplates using an additive‐free, monoalcohol‐directed growth mode. The approach, which sees nanoplates exit the growth solution with clean, unobstructed, ready‐to‐use surfaces, provides an attractive alternative to existing substrate‐based nanoplate growth modes reliant on the use of difficult‐to‐remove surfactants. Through wavelength‐dependent characterization, in combination with an experimental design that decouples the photochemical and growth steps, it is decisively shown that photoexcitations within the growth solution are the key driver of nanoplate growth. DFT calculations reveal the facet‐selective mechanism: adsorbed Cl strengthens methanol binding selectively on Au(111), synergistically driving {111}‐faceted nanoplate growth. These findings, hence, demonstrate that shape‐control is indeed achievable in a monoalcohol‐based synthesis, advance the mechanistic understanding needed to facilitate this outcome, and provide a route for eliminating surface‐contamination issues that can hamper the downstream processing and application of substrate‐based Au nanoplates.

## Conflicts of Interest

The authors declare no conflicts of interest.

## Supporting information




**Supporting File**: smtd70731‐sup‐0001‐SuppMat.pdf.

## Data Availability

The data that supports the findings of this study are available in the supplementary material of this article.
