# Supplementary material for: Monoalcohol‐Directed Shape Control in a Surfactant‐Free Gold Nanoplate Synthesis
Source: Small Methods. 2026 May 20;10(17):e70731. doi: 10.1002/smtd.70731 (PMC13555630; doi:10.1002/smtd.70731)
Supplement: Supplementary file 1 [file SMTD-10-e70731-s001.pdf]

Supporting Information

Monoalcohol-Directed Shape Control in a Surfactant-Free Gold Nanoplate Synthesis

Brendan D. Nieukirk,[a] Shuiyi Zhang,+[b] Runze Tang,+[c] Kaikui Xu,[c] Matthew R. Rosenberger,[c] Robert A. Hughes,[c] Kristen A. Fichthorn,[b,d] Svetlana Neretina*[a,c]*

[a] Department of Chemistry and Biochemistry, University of Notre Dame, Notre Dame, Indiana 46556, United States
[b] Department of Chemical Engineering, The Pennsylvania State University, University Park, Pennsylvania 16802, United States
[c] College of Engineering, University of Notre Dame, Notre Dame, Indiana 46556, United States
[d] Department of Physics, The Pennsylvania State University, University Park, Pennsylvania 16802, United States

* fichthorn@psu.edu (DFT calculations)
* sneretina@nd.edu

Experimental Section

Chemicals and Materials. Au seed arrays were fabricated on two-side polished [0001]-oriented sapphire substrates diced from 100 mm diameter wafers (MTI Corporation) to dimensions of 10 mm × 10.5 mm × 0.65 mm. The metal sputter targets and lithographic materials used in seed fabrication are listed elsewhere.[1] The reagents used in nanoplate synthesis are hydrogen tetrachloroaurate(III) trihydrate ($HAuCl_4 \cdot 3H_2O$, 99.99% trace metal basis, Beantown Chemical), methanol (MeOH, 99.8%, HPLC, Avantor), ethanol (EtOH, absolute, MilliporeSigma), isopropyl alcohol (i-PrOH, 99.8%, HPLC, Sigma-Aldrich), and deionized (DI) water (BDH Chemicals). The 1,8-octanedithiol (C8DT) linkers used to form the core−satellite assemblies in **Figure S4** were sourced from Fisher Scientific.

Au Seed Arrays. The procedure used to form Au seed arrays suitable for nanoplate growth are described in prior reports along with extensive characterization.[1–5] Briefly stated, seed fabrication proceeds through the following steps: (i) a bilayer resist comprised of a lift-off and moldable resist is spin-coated onto a sapphire substrate, (ii) nanoimprint lithography is used to emboss an arrayed pattern of cylindrical holes into the moldable resist using a commercially available silicon stamp, (iii) reactive ion etching (RIE) is used to uniformly thin the patterned resist until the cylindrical holes expose the substrate surface, (iv) Sb and Au are sputter-deposited onto the sample to thicknesses of 20 and 1.5 nm, respectively, followed by a standard lift-off procedure, (v) the sample, which is now comprised of an array of Au-capped Sb discs is heated to 1020 ºC in a N_2 gas atmosphere to assemble an array of crystalline Au seeds as the sacrificial Sb component is lost to the vapor phase and then cooled to room temperature. Seed arrays were typically cut into two equal pieces where each piece was dedicated to a different experiment.

Nanoplate Synthesis. All syntheses were carried out in air at room temperature in an uncovered, triple-rinsed, single-use, 30 mL polypropylene beaker containing a Pyrex magnetic stir bar (length = 12 mm, diameter = 9.5 mm) rotating at 300 rpm. The synthesis began by adding a monoalcohol (*i.e.*, MeOH, EtOH, i-PrOH) to DI H_2O where the combined volume is 9.2 mL prior to mixing and the proportions are as indicated for the various experiments. It should be noted that no allowances are made for the (i) overall volume contraction and slight heating that occurs upon mixing and (ii) evaporation losses. Control measurements showed that evaporation losses were less than 50 μL/h. Moreover, it should be understood that, while comparisons between the various monoalcohols are made based on equal monoalcohol-to-H_2O volume ratios, the molarities are far from equal (see **Table S1**). Aqueous $HAuCl_4$ (0.8 mL, 10 mM) from a stock solution is then pipetted into the monoalcohol-H_2O mixture, followed by the insertion of the Au seed array that rests at the bottom of the beaker across from the stir bar. Syntheses are either paused for characterization or terminated through the removal of the sample with Teflon-coated tweezers followed by a sequential rinsing procedure in DI H_2O, EtOH, and acetone where the sample remains wet until it is slowly pulled out of acetone as it air-dries. Stir bars are cleaned overnight in *aqua regia* (Hazard: *aqua regia* is highly toxic and corrosive) and triple rinsed in DI water before reuse. Syntheses were carried out where the light environment was varied between room light (500 lux), wavelength-specific LED light (1 W m^{-2} as measured at the surface of the stir plate), and darkness. The apparatus used for wavelength-specific measurements is described in detail elsewhere.[6] It consists of a 3D-printed housing that is placed over a growth solution beaker resting on a stir plate. The housing supports a ring of 3 interchangeable LEDs with downward-directing reflectors and is lined with aluminum foil to promote uniform illumination. For syntheses carried out in the dark, it should be recognized that low-level light exposure of the growth solution is required when removing and reinserting the sample. In general, stray light exposure was minimized by carrying out the syntheses and spectroscopic characterization in a dedicated dark room.

Assemblies Utilizing Dithiol Functionalization. The procedures used to functionalize nanoplates with the C8DT linker and then assemble colloidal nanoparticles onto their surface (*i.e.*, **Figure S4**) are described in a prior report.[7]

Spectroscopic Characterization. The time-dependent LSPR data was extracted from spectra obtained in the 350 to 1100 nm spectral range. Obtaining these spectra required that the sample be removed from the growth solution, rinsed, characterized, and reinserted. The entire procedure lasts approximately 5 min where every effort was made to keep the process consistent from measurement-to-measurement and sample-to-sample perspectives. Additional spectra were sometimes acquired post-synthesis on a NIR spectrophotometer to obtain spectral dependencies up to 2500 nm. In both cases, samples were mounted within the spectrophotometer using 3D-printed holders specifically designed for these experiments where all nanoplate spectra were referenced against a bare sapphire substrate. SEM characterization was routinely performed on samples to confirm that the position of the final LSPR was consistent with the nanoplate size obtained. Growth solutions were spectroscopically characterized in a 0.7 mL cuvette with a 2 mm path length and referenced against an identical empty cuvette.

Instrumentation and Data Analysis. SEM images were obtained using a Helios G4 Ux SEM/FIB workstation (FEI) operating in secondary electron mode. AFM characterization was performed on a Cypher ES AFM (Oxford Instruments) equipped with 160AC-NA MikroMasch OPUS SPM

probes. To clearly visualize the Au lattice (**Figure 1d**), a FFT analysis was performed on the AFM lateral signal raw data. The characteristic FFT peaks were then selected and used for lattice reconstruction, effectively removing background noise and resolving the close-packed atoms. Nanoplate extinction spectra were measured using either a Jasco V-730 UV−vis spectrophotometer or a Jasco V-770 UV-vis/NIR spectrophotometer. An OceanView Flame-S-XR1-ES Miniature Spectrometer was used to obtain the emission intensity spectra of the various LEDs.

Computational Methods

DFT calculations. All DFT calculations were performed using the Vienna *Ab initio* Simulation Package (VASP)[8−10] with the Projected Augmented Wave (PAW) method.[11] The generalized gradient approximation (GGA) with the Perdew-Burke-Ernzerhof (PBE) functional[12] was employed for the exchange-correlation energy. The DFT-D3 method of Grimme[13] was applied to describe long-range van der Waals (vdW) interactions. The energy cutoff for the plane-wave basis set was 450 eV and Monkhorst-Pack grids[14] were used for the k points. A 0.01 $eV/Å$ was used as the atomic force convergence criterion and 10^{-6} eV for the energy convergence criterion. A 6-layer slab was used with the bottom three layers fixed at the bulk Au positions with a calculated lattice constant of 4.11 Å. The calculated lattice constant agrees well with the experimental value of 4.08 Å[15] and falls within the range of calculated values for vdW-corrected DFT.[16] The bulk energy we find here is $\mu_{Au} = -3.88$ eV/atom, in agreement with values from a previous study using various forms of vdW-corrected DFT.[16] A dipole correction was applied in the z direction normal to the slab. To remove unphysical interactions between unit cells, all the configurations in the calculation include a 20 Å vacuum spacing in the z direction. To access a wide range of adsorbate coverages and configurations, various supercell sizes were employed, including (2×2), (2×3), (3×3), (3×4), and (4×4) unit cells, sampled with $(14 \times 14 \times 1)$, $(14 \times 9 \times 1)$, $(9 \times 9 \times 1)$, $(9 \times 7 \times 1)$, and $(7 \times 7 \times 1)$ $\boldsymbol{k}$-point mesh, respectively.

MeOH and Cl binding energies were calculated using

$$E_{bind,MeOH} = E_{Au+Cl\,(+MeOH)\,slab\,i} - E_{MeOH} - E_{slab\,i}\,, \qquad (S1)$$

and

$$E_{bind,Cl} = \frac{E_{Au+MeOH\,slab\,i} - N_{Cl}E_{Cl} - E_{slab\,i}}{N_{Cl}}\,. \qquad (S2)$$

Here, $E_{Au+Cl\,(+MeOH)\,slab\,i}$ is the total energy of a slab containing Cl and possibly one MeOH (in the case that the unit cell contains more than one MeOH) on surface i [$i =$(100) or (111)], E_{MeOH} is the energy of gas-phase MeOH, $E_{slab\,i}$ is the total energy of slab i containing the full number of Cl and MeOH, $E_{Au+MeOH\,slab\,i}$ is the total energy of a slab containing MeOH, N_{Cl} is the number of adsorbed Cl, and E_{Cl} is the energy of gas-phase Cl. Here, we use $E_{Cl} = \frac{1}{2}E_{Cl_2}$.

CI-NEB calculations were performed using VASP with VTST, and each calculation employed 3 or 5 intermediate images between the initial and final states. The initial and final state geometries were fully optimized with the same convergence criterion mentioned above. The spring constant was set to 5.0 $eV/Å$, and all CI-NEB calculations are converged when the force on each atom was less than 0.02 $eV/Å$. Additional details for all of these calculations are included in the Supporting Information (see **Figures S13−S16**, and **Table S3**).

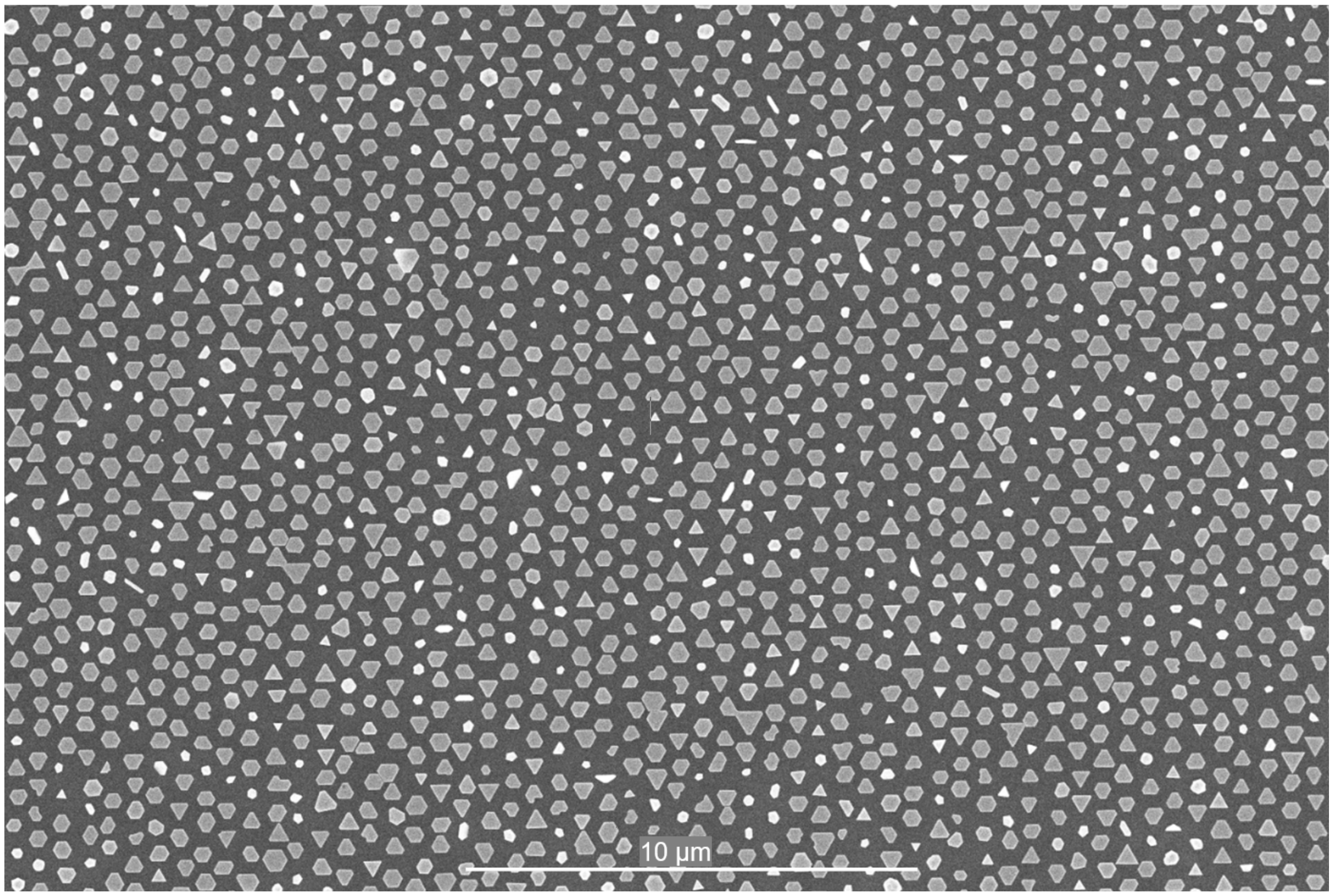

Figure S1. SEM image showing a large-area nanoplate array formed using a 20% MeOH growth solution. The nanoplate yield is 81%.

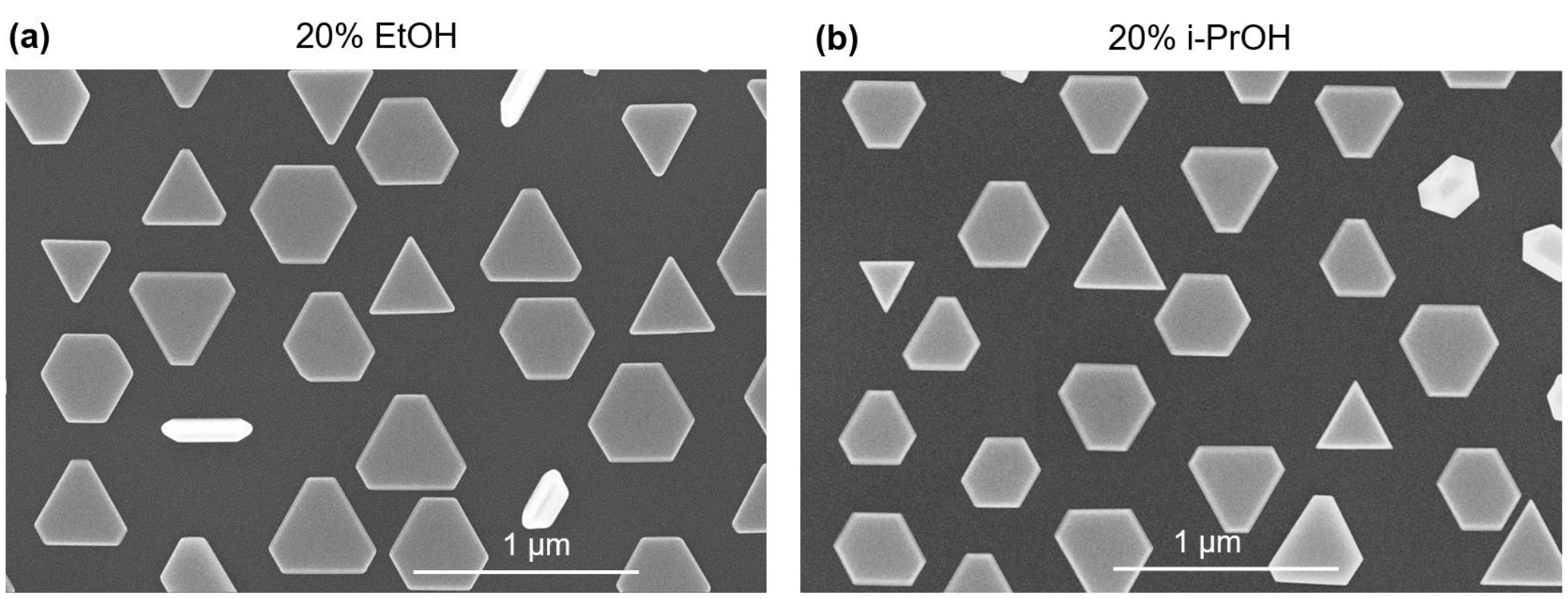

Figure S2. SEM images of the nanoplate arrays formed under room light using 20% (a) EtOH and (b) i-PrOH growth solutions.

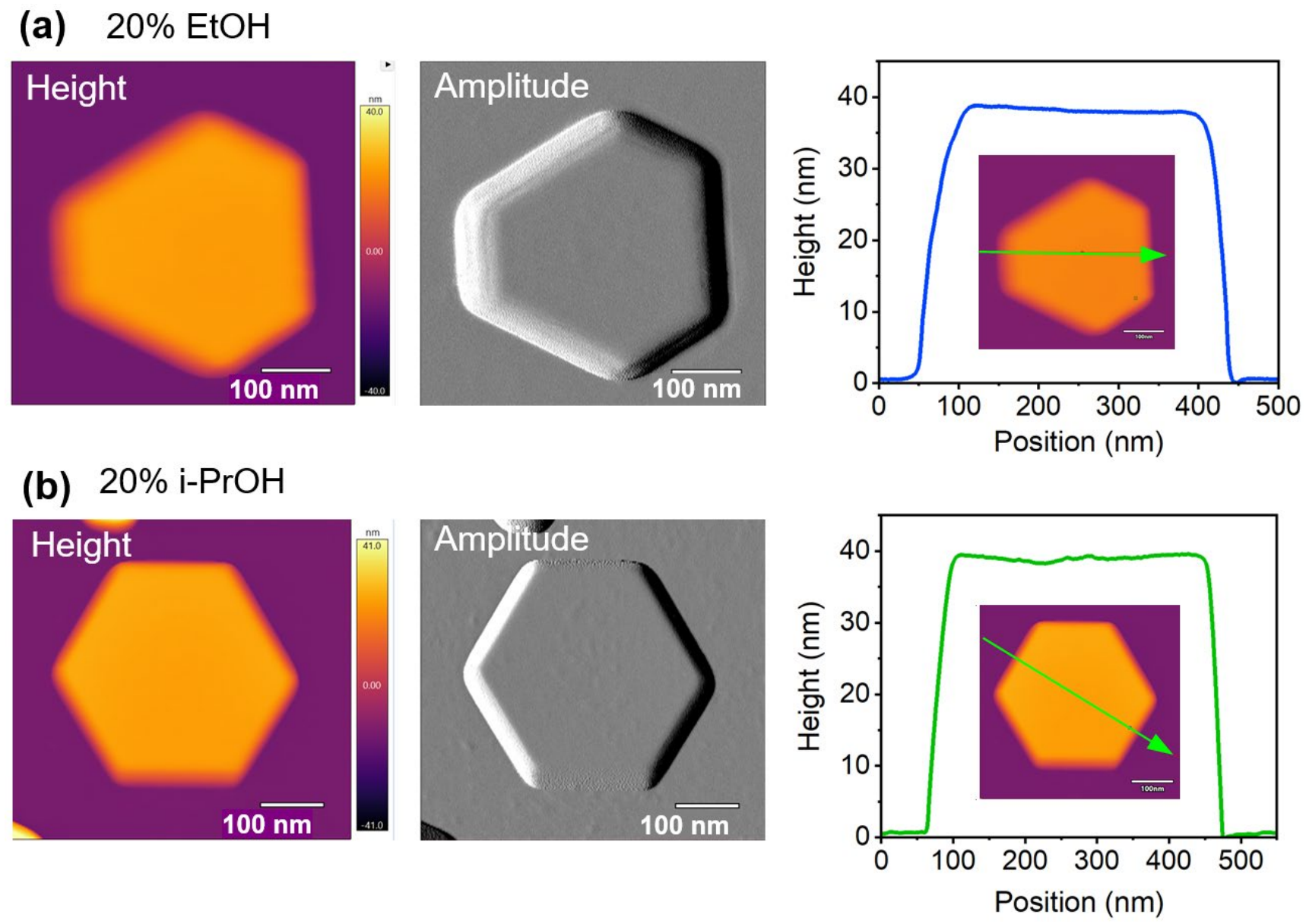

Figure S3. AFM height and amplitude images as well as the associated line scans for nanoplates grown under room light using 20% (a) EtOH and (b) i-PrOH growth solutions.

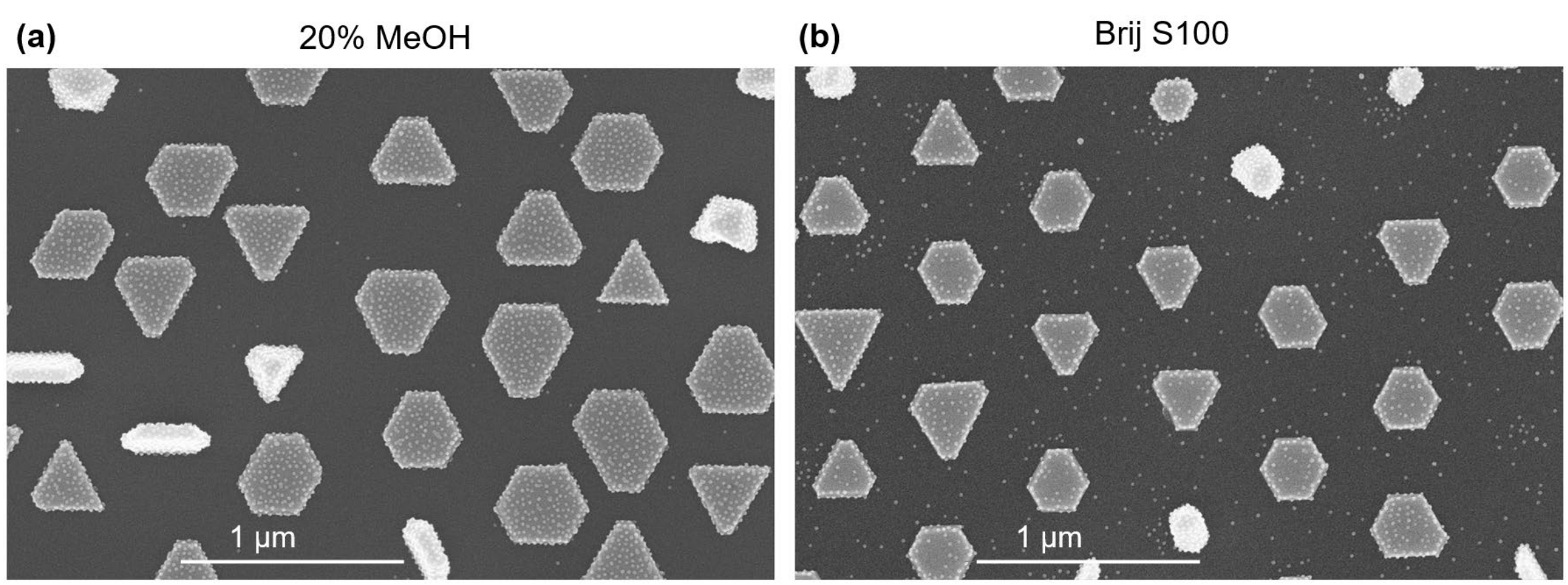

Figure S4. Colloidal Au nanoparticles that have been assembled onto dithiol-functionalized Au nanoplates derived from (a) surfactant-free monoalcohol-based and (b) surfactant-based syntheses. The resulting core−satellite assemblies reveal that the Au surfaces obtained for the surfactant-free case are amenable to the dithiol functionalization needed for assembly whereas those obtained using surfactants are substandard.

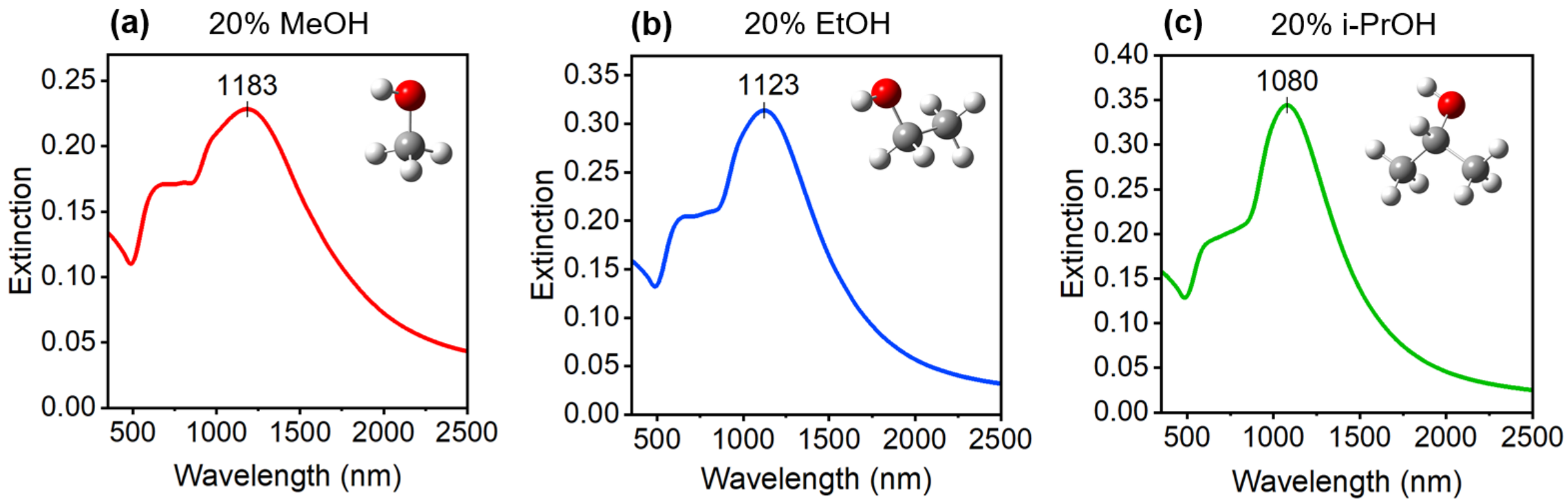

Figure S5. Extinction spectra of Au nanoplates formed under room light in 20% (a) MeOH, (b) EtOH, and (c) i-PrOH growth solutions.

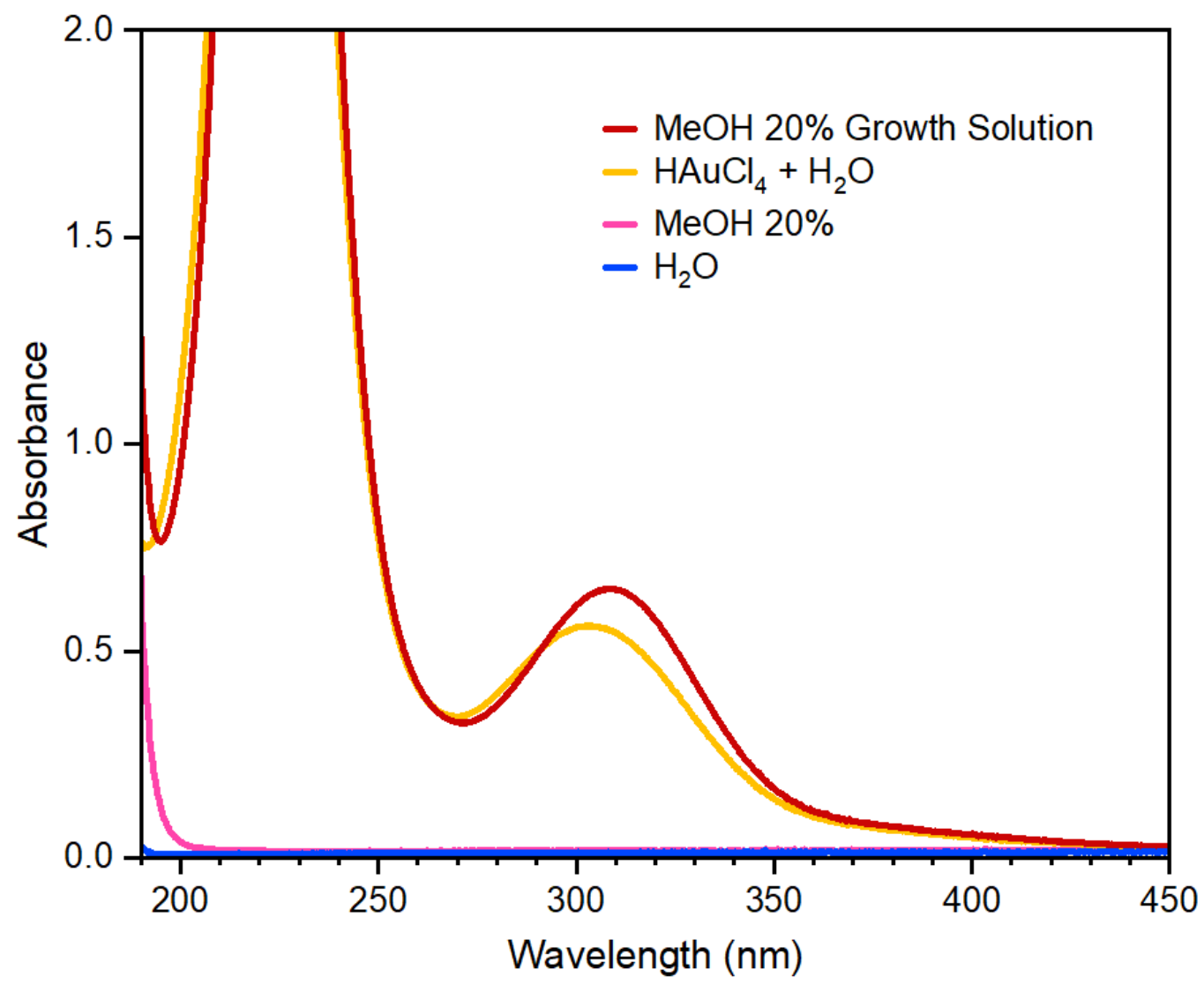

Figure S6. Absorbance spectra for the 20% MeOH growth solution and growth solutions where individual components are excluded and replaced with equivalent volumes of H_2O. The data reveal that the absorbance peaks within the growth solution are largely due to $HAuCl_4$; however, the inclusion of MeOH results in a red shift in the 310 nm peak that is accompanied by an increase in intensity. The H_2O and MeOH components, by themselves, show no appreciable absorption at wavelengths corresponding to the visible spectrum.

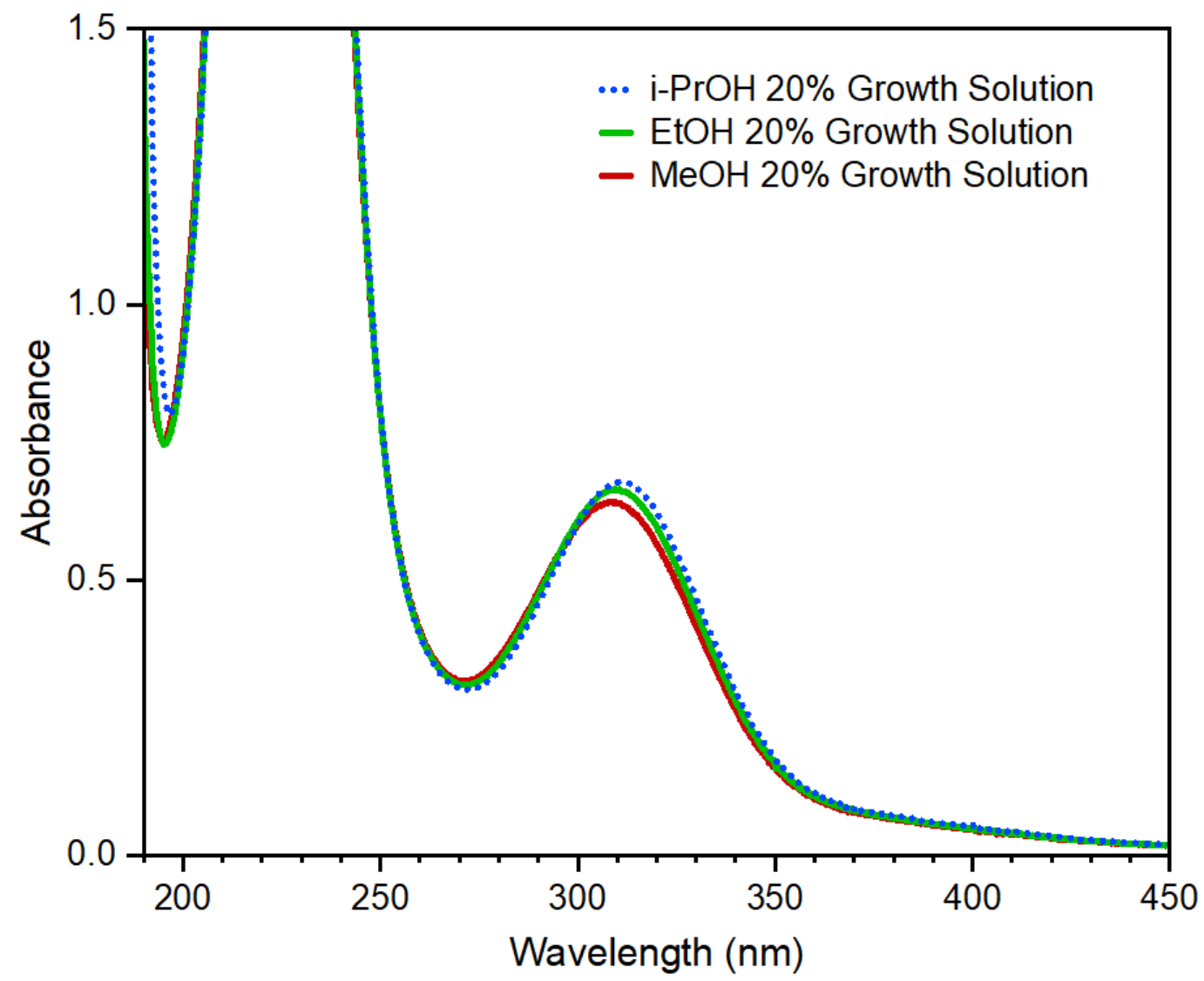

Figure S7. Comparison of the absorbance spectra for growth solutions containing MeOH, EtOH, and i-PrOH at the 20% level by volume. It should be noted that there is a high degree of spectral overlap, indicating that each absorbs nearly identical amounts of light when similarly illuminated.

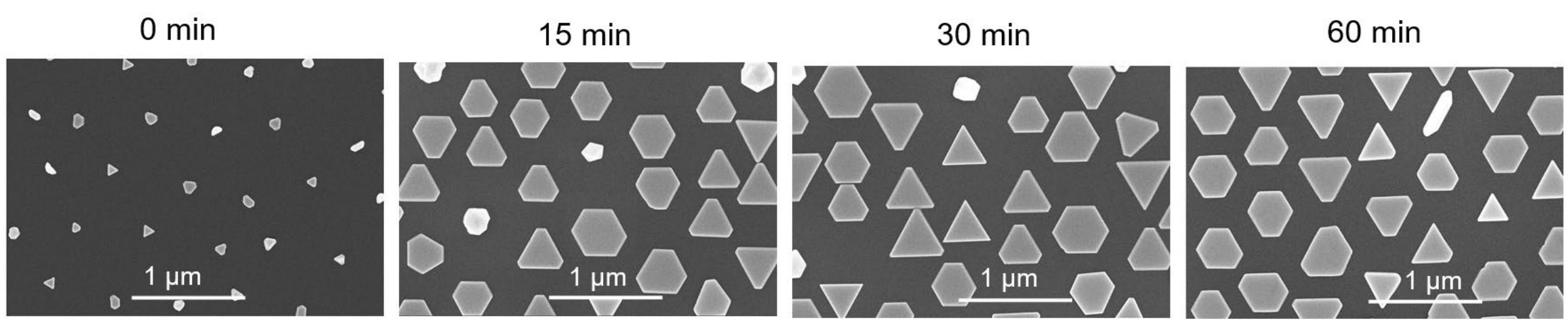

Figure S8. SEM images of Au nanoplates derived from syntheses where the growth solution was "optically primed" with blue light for various times, followed by seed insertion and growth in the dark. The data confirm that, apart from the 0 min result where the growth solution saw no light, the nanoplate growth mode is consistent in terms of the nanoplate morphology realized.

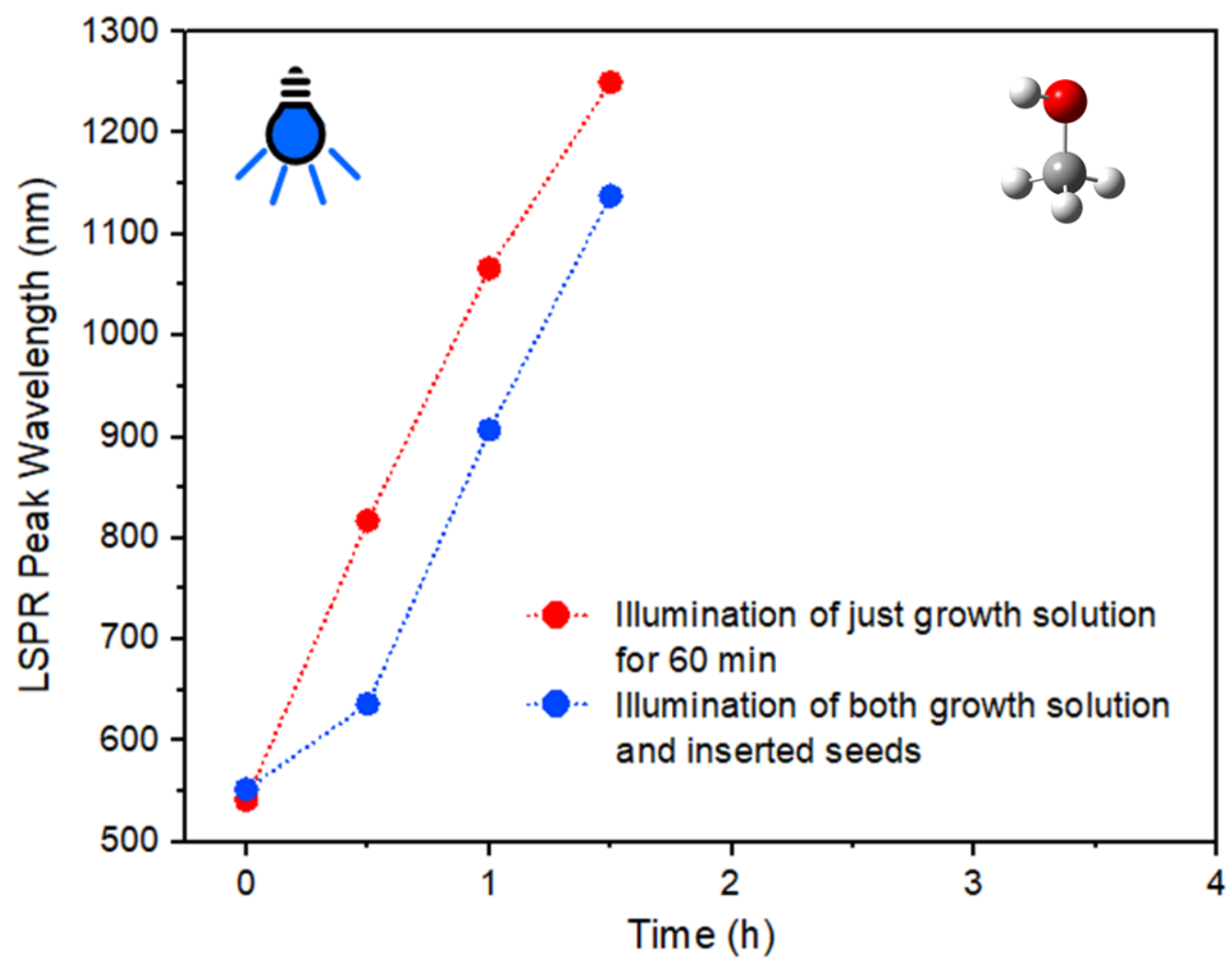

Figure S9. Comparison of the nanoplate LSPR peak wavelength *versus* time data for the cases where the growth solution is illuminated with blue light for 60 min prior to seed insertion and grown in the dark (red curve) and where seed insertion and illumination commence at the same time (blue curve). The faster growth rate observed for the pre-illumination case is likely attributable to the build-up of reduced Au atoms within the growth solution that are readily available for deposition once the seeds are inserted. In contrast, simultaneous illumination requires time to achieve the same build-up of reduced Au.

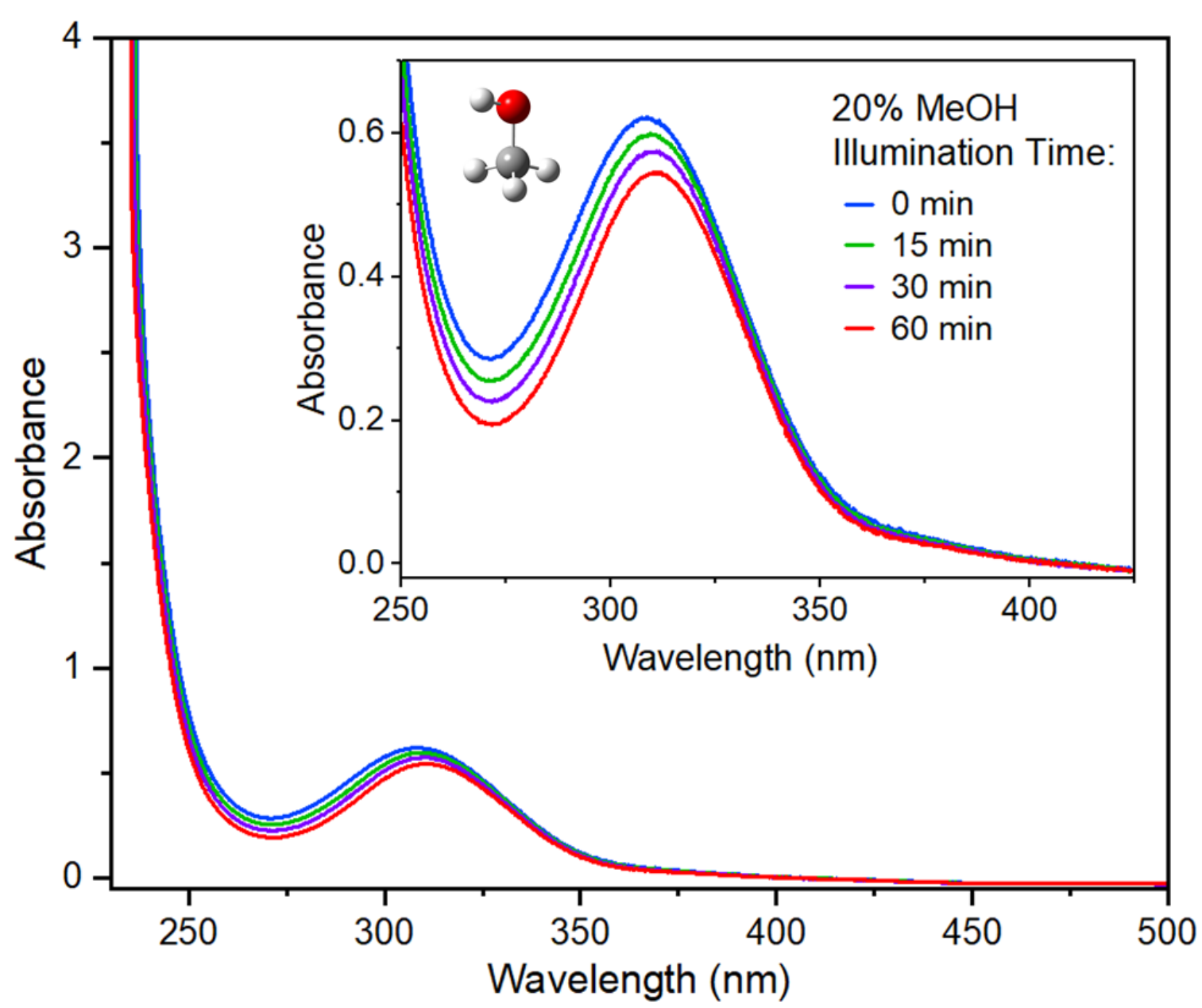

Figure S10. Comparison of the absorbance spectra for growth solutions containing 20% MeOH that were illuminated with blue light for 0, 15, 30, and 60 min. The gradual reduction in the magnitude of the absorbance peak associated with $AuCl_4^-$ provides evidence that the Au precursor is being consumed, likely through a photoredox reaction yielding zero-valent Au atoms.

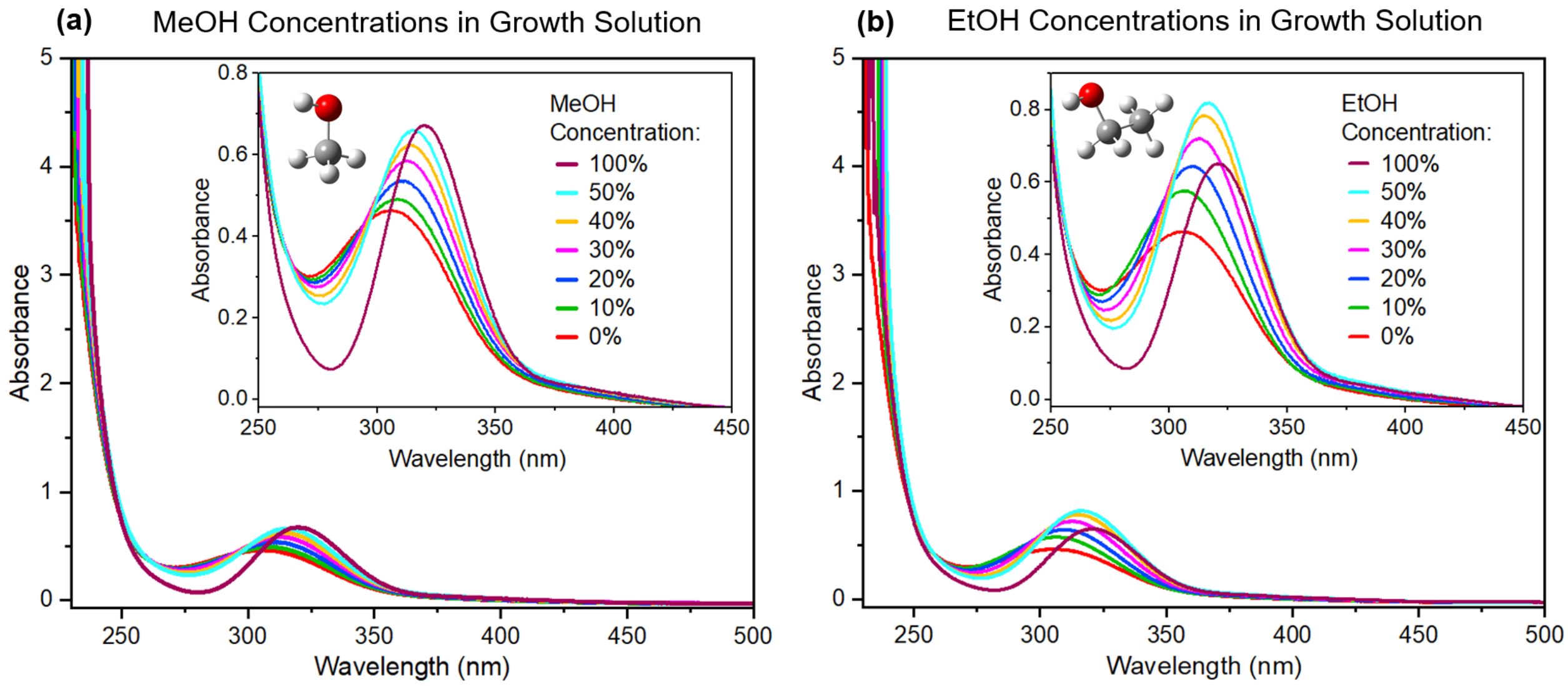

Figure S11. Absorbance spectra for nanoplate growth solutions with (a) MeOH and (b) EtOH concentrations ranging from 0% to 100%. The data reveal that the absorbance peak red shifts and intensifies as the monoalcohol concentration is increased. Given that the 20% concentration gives rise to the fastest growth rate and 100% the slowest, it can be concluded that this difference is not attributable to the growth solution absorbance because this factor, by itself, would predict that the 100% concentration would grow the fastest under blue light illumination.

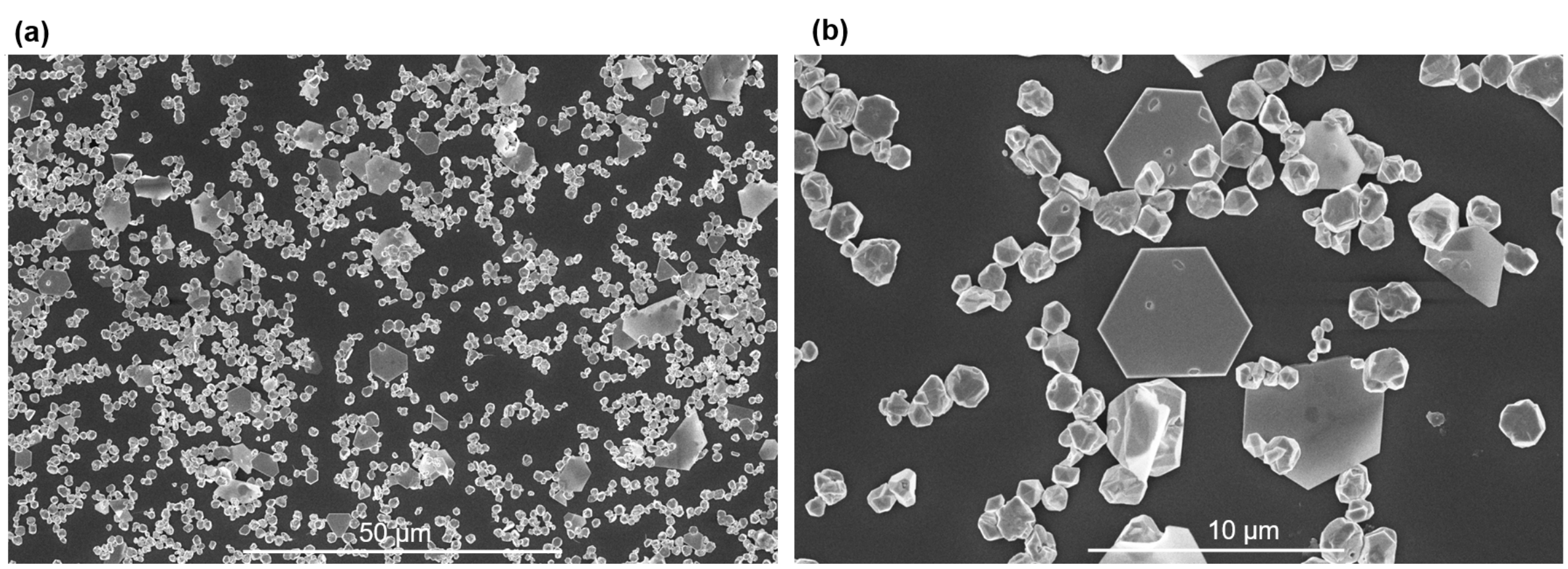

Figure S12. (a) Low- and (b) high-magnification SEM images of the colloids that form within a 20% MeOH growth solution when exposed to blue light for 48 h.

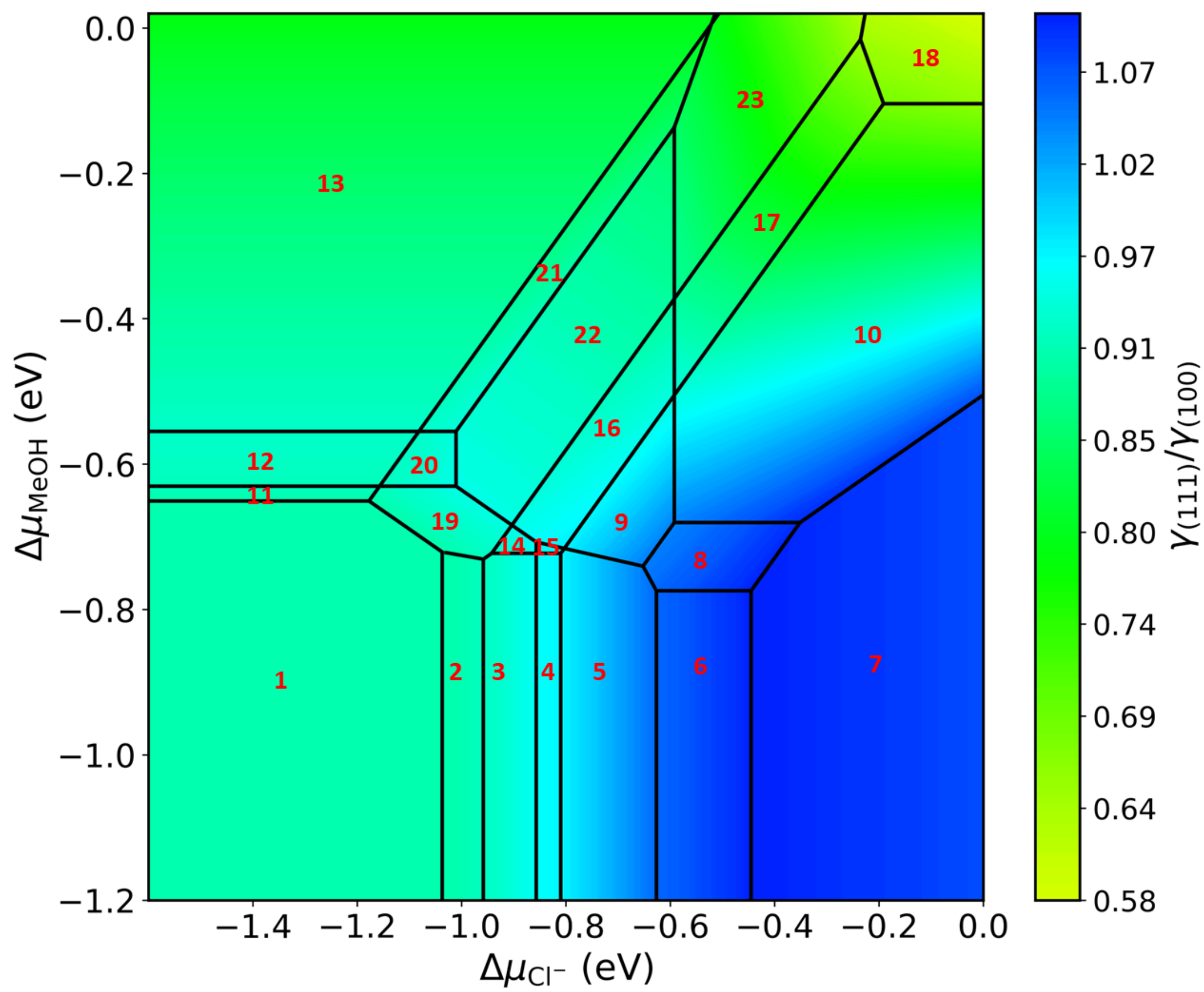

Figure S13. Full phase diagram for MeOH and Cl co-adsorption on Au(100) and Au(111). The MeOH and Cl coverages in each region are shown in the table below.

Region	Au(100)		Au(111)	
	θ_{Cl}	θ_{MeOH}	θ_{Cl}	θ_{MeOH}
1	0	0	0	0
2	1/8	0	0	0
3	1/3	0	0	0
4	1/3	0	1/9	0
5	1/2	0	1/9	0
6	1/2	0	1/3	0
7	1/2	0	1/2	0
8	1/2	0	1/3	1/6
9	1/2	0	1/6	1/3
10	1/2	0	1/3	1/3
11	0	1/2	0	0
12	0	1/2	0	1/3
13	0	1/2	0	1/2
14	1/3	1/6	0	0
15	1/3	1/6	1/9	0
16	1/3	1/6	1/6	1/3
17	1/3	1/6	1/3	1/3
18	1/2	1/4	1/3	1/3
19	1/3	1/6	0	0
20	1/6	1/3	0	1/3
21	1/6	1/3	0	1/2
22	1/6	1/3	1/6	1/3
23	1/6	1/3	1/3	1/3

The calculated binding energies of alcohols and alkoxides relevant to experiments are shown in **Figure S14**. The binding energy of species X is given by

$$E_{bind,X} = \frac{1}{N_X}[(E_{slab} + N_X E_X^*) - E_{slab+X}], \quad (S3)$$

where E_{slab+X} is the energy of an optimized Au surface containing adsorbed species X, E_X^* is the energy of species X in vacuum, N_X is the number of adsorbed X, and E_{slab} is the total energy of a bare Au(111) or Au(100) slab. In all cases, the alkoxides bind significantly more strongly than their corresponding alcohols. Moreover, alkoxide binding is stronger on Au(100) than on Au(111), and a similar facet-dependent trend is observed for alcohol adsorption, except for isopropanol. This similarity further justifies our choice of methanol as a model monoalcohol, as methanol and methoxy exhibit adsorption trends that are representative of other alcohol/alkoxide pairs.

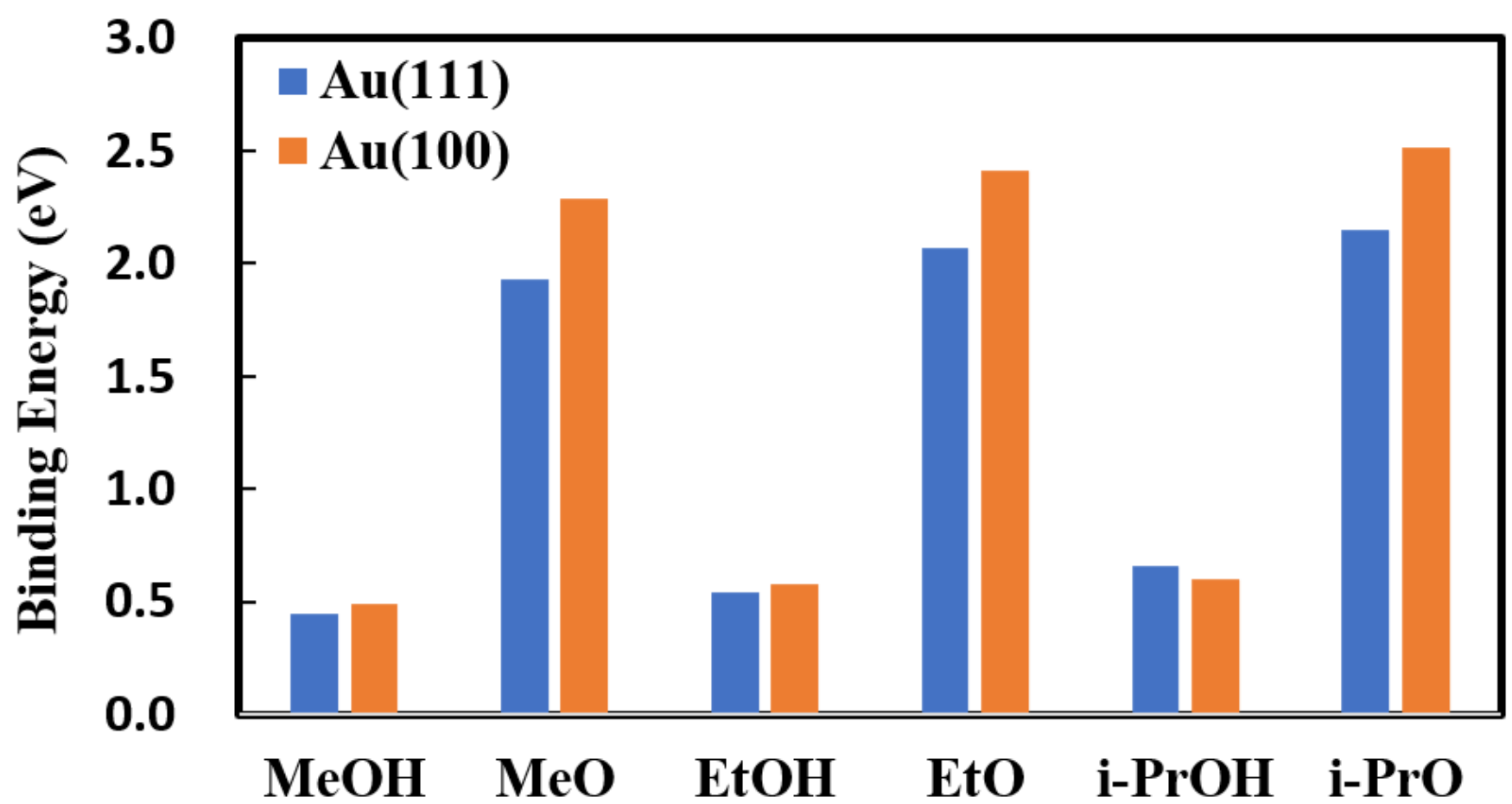

Figure S14. Binding energies of alcohols and alkoxides on Au(111) and Au(100) at a coverage of 1/9 ML.

In addition to comparing the binding energies of methanol and methoxy, an analysis of the respective electronic interactions and van der Waals interactions at a fixed coverage of 1/9 ML was carried out. The results, shown in **Figure S15**, indicate that methanol adsorption is predominantly governed by van der Waals interactions, whereas methoxy binding is dominated by electronic interactions, suggesting that the weakly bound methanol is less effective as a capping agent.

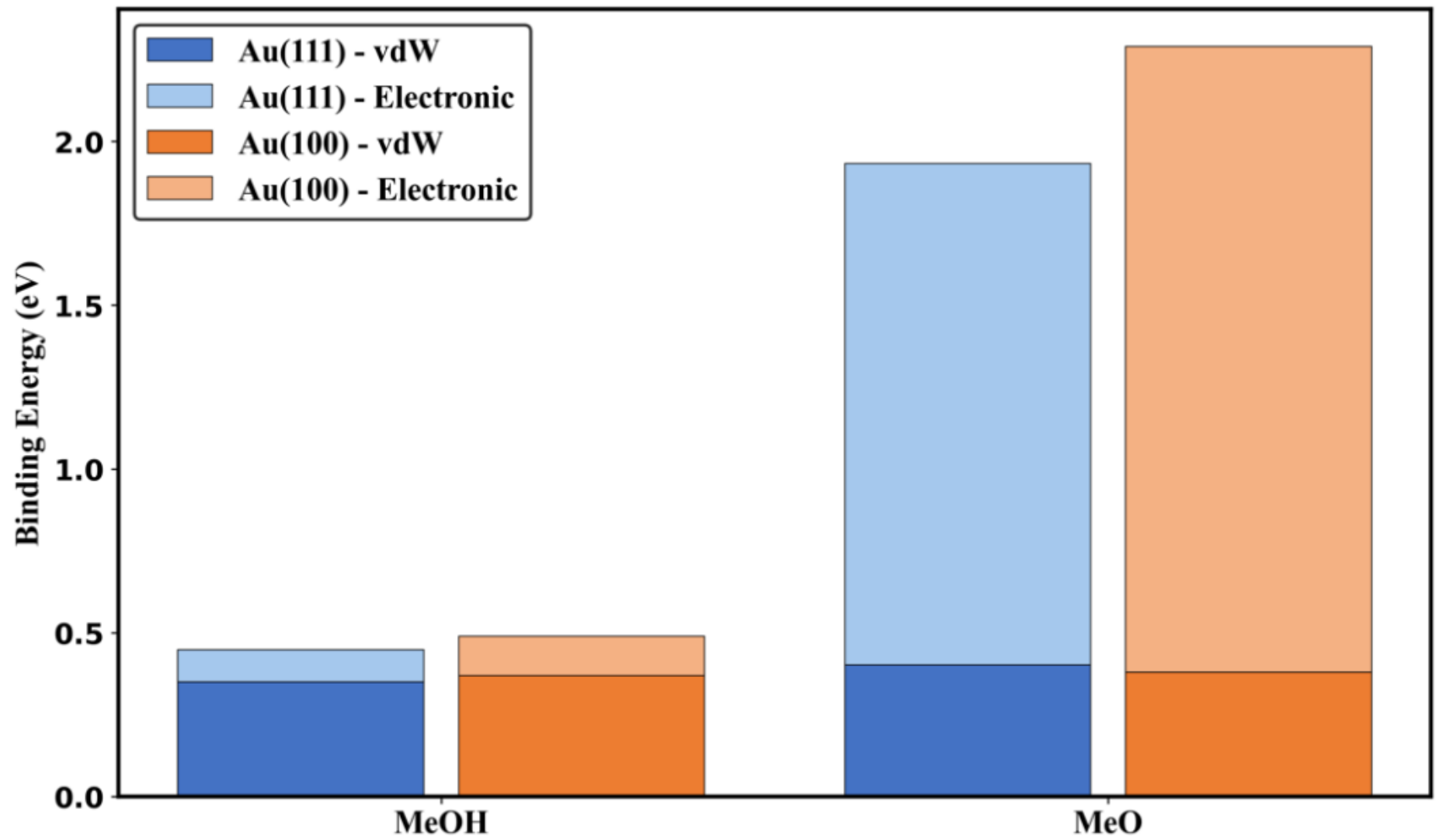

Figure S15. Contributions to methanol and methoxy binding energy from van der Waals (vdW) and electronic interactions at coverage of 1/9 ML.

Figure S16 shows the convergence test with respect to the plane-wave energy cutoff. Single-point calculations were performed for bulk Au using a four-atom primitive cell with a fixed 20 × 20 × 20 Morkhorst-Pack ***k***-point mesh. The *y*- axis, ΔE, is the energy difference between two consecutive cutoff values (point ***N*** and ***N-1***). Based on the test, an energy cutoff of 450 eV was selected, as highlighted by the red circle.

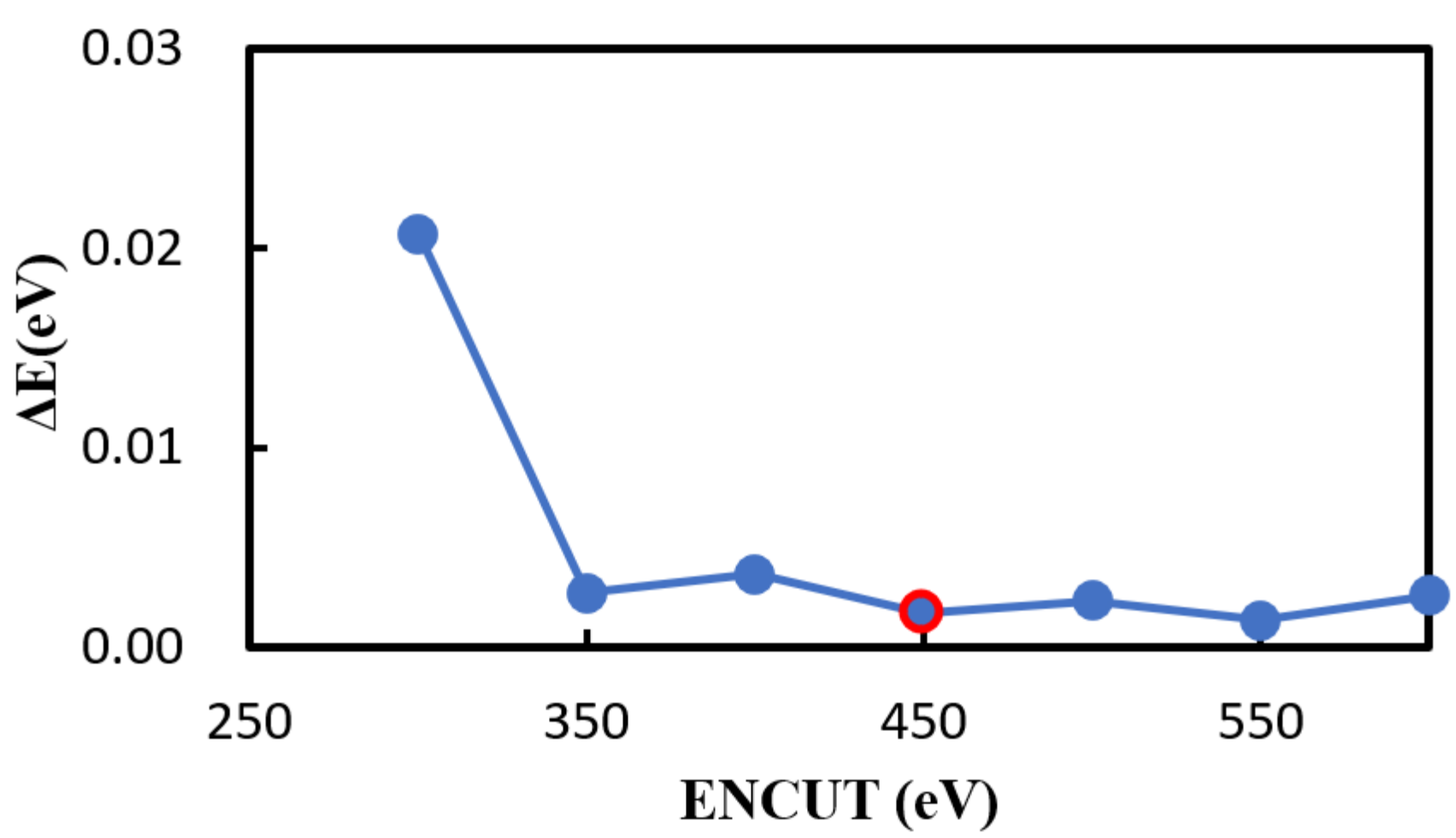

Figure S16. Convergence test of energy cutoff (encut).

Table S1. Listing of the monoalcohol-to-H_2O volume ratios and the associated molarity for the various growth solutions used in the current study.

	Molarity of Alcohol in Solution ($mol*L^{-1}$)		
**% v/v**	**MeOH**	**EtOH**	***i*-PrOH**
1	0.25	0.17	0.13
5	1.23	0.85	0.65
20	4.91	3.41	2.61
25	6.14	4.26	3.27
30	7.37	5.11	3.92
40	9.82	6.82	5.22
100	24.56	17.04	13.06

Table S2. Relevant physicochemical properties for the three monoalcohols used in this study**.**

	Mass ($g*mol^{-1}$)	Density @ 25 °C ($g*mL^{-1}$)	Viscosity @ 25 °C (mPa*s)	Dipole Moment (D)	pKa
**MeOH**	32.042	0.787[a]	0.545[a]	2.61[b]	15.5[c]
**EtOH**	46.069	0.785[a]	1.082[a]	2.56[b]	15.9[c]
***i*-PrOH**	60.096	0.785[d]	2.038[d]	2.71[b]	17.1[e]

a. González, B.; Calvar, N.; Gómez, E.; Domínguez, Á. Density, Dynamic Viscosity, and Derived Properties of Binary Mixtures of Methanol or Ethanol with Water, Ethyl Acetate, and Methyl Acetate at T = (293.15, 298.15, and 303.15) K. *J. Chem. Thermodyn.* **2007**, *39*, 1578−1588.

b. Jorge, M.; Gomes, J. R. B.; Barrera, M. C. The Dipole Moment of Alcohols in the Liquid Phase and in Solution. *J. Mol. Liq.*, **2022**, *356*, 119033.

c. Ballinger, P.; Long, F. A. Acid Ionization Constants of Alcohols. II. Acidities of Some Substituted Methanols and Related Compounds1,2. *J. Am. Chem. Soc.* **1960**, *82*, 795–798.

d. Haynes, W. M. *CRC Handbook of Chemistry and Physics*, 95th ed.;CRC Press: Boca Raton, FL, **2014**.

e. Serjeant, E. P.; Dempsey, B. *Ionisation Constants of Organic Acids in Aqueous Solution*; Permagon: Oxford, **1979**.

Table S3. Convergence test on the binding energy of methoxy on Au surfaces with respect to the ***k***-point mesh.

***k*-point mesh**	**surface supercell**	**$E_{binding,MeO}$ (eV)**
$10 \times 10 \times 1$	Au(100)-(2 × 2)	2.22
$11 \times 11 \times 1$	Au(100)-(2 × 2)	2.21
$12 \times 12 \times 1$	Au(100)-(2 × 2)	2.22
$13 \times 13 \times 1$	Au(100)-(2 × 2)	2.22
$14 \times 14 \times 1$	Au(100)-(2 × 2)	2.21
$15 \times 15 \times 1$	Au(100)-(2 × 2)	2.21
$14 \times 7 \times 1$	Au(100)-(2 × 3)	2.28
$14 \times 8 \times 1$	Au(100)-(2 × 3)	2.27
$14 \times 9 \times 1$	Au(100)-(2 × 3)	2.26
$14 \times 10 \times 1$	Au(100)-(2 × 3)	2.26
$14 \times 11 \times 1$	Au(100)-(2 × 3)	2.26

References

[1] R. D. Neal, Z. R. Lawson, W. J. Tuff, et al., "Large-Area Periodic Arrays of Atomically Flat Single-Crystal Gold Nanotriangles Formed Directly on Substrate Surfaces" *Small* **18,** (2022): 2205780.

[2] S. D. Golze, R. A. Hughes, S. Rouvimov, R. D. Neal, T. B. Demille, S. Neretina, "Plasmon-Mediated Synthesis of Periodic Arrays of Gold Nanoplates using Substrate-Immobilized Seeds Lined with Planar Defects" *Nano Letters* **19,** (2019): 5653.

[3] Z. R. Lawson, K. Xu, C. Boukouvala, et al., "Large-Area Arrays of Epitaxially-Aligned Silver Nanotriangles Seeded by Gold Nanostructures" *Materials Chemistry Frontiers* **8,** (2024): 2149.

[4] T. B. Demille, R. D. Neal, A. S. Preston, et al., "Epitaxially Aligned Single-Crystal Gold Nanoplates Formed in Large-Area Arrays at High Yield" *Nano Research* **15,** (2022): 296.

[5] S. D. Golze, R. A Hughes, E. Menumerov, S. Rouvimov, S. Neretina, "Synergistic Roles of Vapor- and Liquid-Phase Epitaxy in the Seed-Mediated Synthesis of Substrate-Based Noble Metal Nanostructures" *Nanoscale* **13,** (2021): 20225.

[6] Z. R. Lawson, L. Ciambriello, B. D. Nieukirk, et al., "Light-Mediated Growth of Gold Nanoplates Carried out in Total Darkness" *ACS Nano* **19,** (2025): 9378.

[7] R. Tang, R. A. Hughes, W. J. Tuff, A. Corcoran, S. Neretina, "Rapid Formation of Gold Core-Satellite Nanostructures Using Turkevich-Synthesized Satellites and Dithiol Linkers: The Do's and Don'ts for Successful Assembly" *Nanoscale Advances* **6**, (2024): 3632.

[8] G. Kresse, J. Hafner, "Ab Initio Molecular-Dynamics Simulation of the Liquid-Metal-Amorphous-Semiconductor Transition in Germanium" *Physical Review B* **49**, (1994): 14251.

[9] G. Kresse, J. Hafner, "Ab Initio Molecular Dynamics for Liquid Metals" *Physical Review B* **47**, (1993): 558.

[10] G. Kresse, J. Furthmüller, "Efficient Iterative Schemes for Ab Initio Total-Energy Calculations Using a Plane-Wave Basis Set" *Physical Review B* **54**, (1996): 11169.

[11] P. E. Blöcl, "Projector Augmented-Wave Method" *Physical Review B* **50**, (1994): 17953.

[12] J. P. Perdew, K. Burke, M. Ernzerhof, "Generalized Gradient Approximation Made Simple" *Physical Review Letters* **77**, (1996): 3865.

[13] S. Grimme, J. Antony, S. Ehrlich, H. Krieg, "A Consistent and Accurate Ab Initio Parametrization of Density Functional Dispersion Correction (DFT-D) for the 94 Elements H-Pu" *Journal of Chemical Physics* **132**, (2010): 154104.

[14] H. J. Monkhorst, J. D. Pack, "Special Points for Brillouin-Zone Integrations" *Physical Review B* **13**, (1976): 5188.

[15] *CRC Handbook of chemistry and Physics: A Ready-Reference Book of Chemical and Physical Data, 76th ed* (Ed.: D. R. Lide), CRC Press, Boca Raton, (1995).

[16] A. Ambrosetti, P. L. Silvestrelli, "Cohesive Properties of Noble Metals by van der Waals-Corrected Density Functional Theory: Au, Ag, and Cu as Case Studies" *Physical Review B* **94**, (2016): 045124.
